# QM/MM Free Energy Calculations of IRE1 Reveal a Unique Protonation State of the Catalytic Lys599

**DOI:** 10.1002/jcc.70288

**Published:** 2025-12-04

**Authors:** Antonio Carlesso, Paolo Conflitti, Sayyed Jalil Mahdizadeh, Giuseppe Deganutti, Leif A. Eriksson, Vittorio Limongelli

**Affiliations:** ^1^ Department of Pharmacology, Sahlgrenska Academy University of Gothenburg Gothenburg Sweden; ^2^ Euler Institute, Faculty of Biomedical Sciences Università della Svizzera italiana (USI) Lugano Switzerland; ^3^ Department of Chemistry and Molecular Biology University of Gothenburg Gothenburg Sweden; ^4^ Centre for Health and Life Sciences Coventry University Coventry UK

**Keywords:** catalytic lysine, IRE1, metadynamics, protonation state, QM/MM

## Abstract

Inositol Requiring Enzyme 1 (IRE1) is a bifunctional serine/threonine kinase and endoribonuclease identified as therapeutic target in multiple diseases. Inspired by the recent work on the assessment of lysine and cysteine reactivities, we present a simple and intuitive protocol for the assessment of reactive lysine, while characterizing a unique protonation state of Lys599 located in the kinase domain. Using Quantum Mechanics/Molecular Mechanics (QM/MM) calculations, QM/MM well‐tempered metadynamics simulations (QM/MM WT‐MetaD), and classical Molecular Dynamics (MD), we have investigated inhibitor binding in three different states of the IRE1 kinase: (i) DFG‐in/αC‐in (DICI) conformation; (ii) the DFG‐out/αC‐out (DOCO) conformation, and (iii) the DFG‐in/αC‐out (DICO) conformation. Our findings reveal a unique proton transfer from the sidechain of the β3‐strand Lys599 to Glu612 of the αC‐helix. Our results allow for accurately defining the geometry of the hydrogen bonds occurring in the IRE1 kinase active state and distinguishing structurally closely related inactive states by analyzing the formation/disruption of crucial hydrogen bonds in the Lys599‐Glu612‐Asp711 triad. Our work prompts further studies in IRE1 and other kinases to characterize possibly conserved drug binding mechanisms that might lead to a novel structural paradigm in kinase drug discovery.

## Graphical abstract

Different conformations at the IRE1 kinase site, imposed by different inhibitors, lead to changes in interactions between the key catalytic residues which in turn stabilize Lysine 599 in its neutral (deprotonated) form.

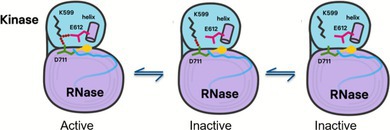

## 1 Introduction

Protein kinases are enzymes that catalyze the transfer of the ATP γ‐phosphate to the side chains of amino acids like serine, threonine, and tyrosine in substrate proteins [1]. The key functional role of protein kinases in cell growth, motility, and death has endorsed the kinase protein family, loosely referred to as the kinome, as prominent drug targets [1].

**FIGURE 1:**
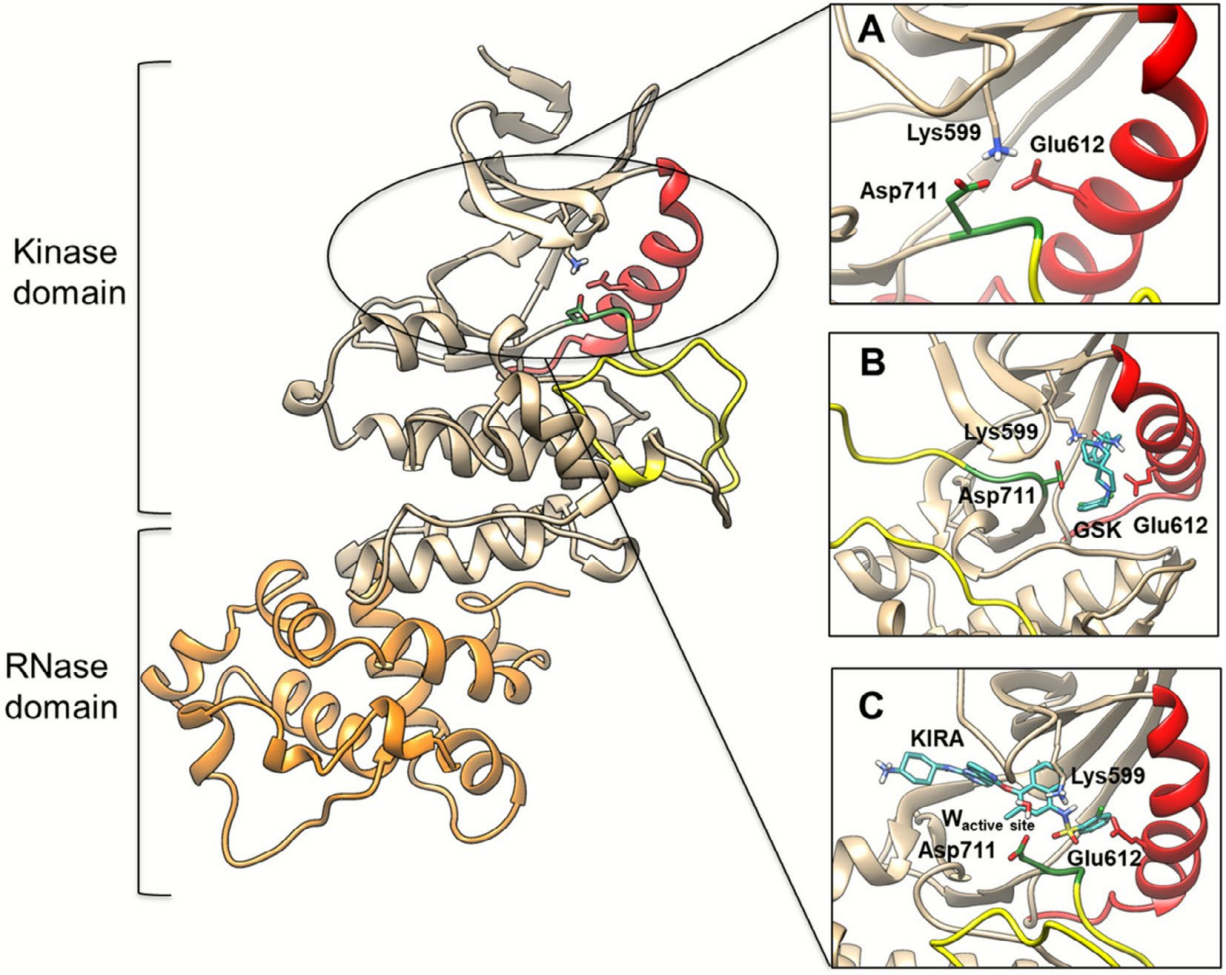
Ribbon diagram structures of IRE1 in: (A) DICI conformation (PDB code 6W3B, active conformation [3]), (B) DOCO conformation (PDB code 4YZ9, inactive conformation [4]) co‐crystallized with 2,7‐diazaspiro[4.5]decane inhibitor GSK2850163 [5] and (C) DICO conformation (PDB code 4U6R, inactive conformation [6, 7]) co‐crystallized with a sulfonamide inhibitor [5]. The αC‐helix, DFG motif, and activation loop are shown in red, green, and yellow, respectively.

From a structural point of view, eukaryotic protein kinase domains are comprised of a β‐strand‐rich N‐lobe (β1–β5) and an α‐helical C‐lobe connected by a flexible hinge region, forming the ATP‐binding site [2] (Figure 1). The αC helix at the N‐lobe contains a highly conserved Glu residue, which forms a salt bridge with the catalytic Lys on the β3 strand in the active form of the kinase (i.e., αC‐in conformation [8]). Adjacent to the active site, a characteristic and conserved “DFG” sequence (Asp, Phe, and Gly) marks the beginning of the activation loop, typically 20–30 residues long. The DFG motif can adopt an active‐like conformation—i.e., DFG‐in—where the Asp forms a salt bridge with the catalytic Lys, while in the so‐called DFG‐out conformation, the Asp residue is far from the catalytic Lys. In summary, interactions between the catalytic Lys and Glu in the αC helix, and between Lys and Asp in the “DFG” motif, play key roles in the protein kinases' catalytic activity. Such molecular interactions characterize the active and inactive states of the kinase, specifically the DFG‐in/αC‐in (DICI) conformation (active conformation), the DFG‐in/αC‐out (DICO) (inactive conformation) and the DFG‐out/αC‐out (DOCO) conformation (inactive conformation) [9].

Such classification of protein kinase structures, albeit simplistic, is useful in guiding structure‐based drug design [10, 11]. However, a deeper insight into the structural mechanism of protein kinases is in great demand [2, 12, 13, 14, 15, 16]. In the present work, *in silico* QM/MM calculations, QM/MM well‐tempered metadynamics (QM/MM WT‐MetaD), and classical Molecular Dynamics (MD) simulations were performed on three different states of the human Inositol Requiring Enzyme 1 (IRE1) kinase enzyme. The IRE1 kinase represents a relevant therapeutic target for various diseases including cancer, and degenerative, inflammatory, and metabolic pathologies [5]. IRE1 is involved in the Unfolded Protein Response (UPR), promoting either adaptation or apoptosis [17, 18] by means of the following steps: (a) oligomerization of IRE1, (b) kinase domain autophosphorylation, (c) activation of the IRE1α RNase domain and (d) final cleavage of the transcription factor X‐box‐binding protein 1 (XBP1), a key actor in the UPR initiation [18, 19, 20].

The states explored herein include (i) the DICI conformation (PDB code 6W3B, active conformation [3]; “apo”); (ii) the DOCO conformation (PDB code 4YZ9, inactive conformation [4] co‐crystallized with non‐covalent 2,7‐diazaspiro[4.5]decane inhibitor GSK2850163 [5]; “GSK”); and (iii) the DICO conformation (PDB code 4U6R, inactive conformation [6, 7] co‐crystallized with a non‐covalent sulfonamide inhibitor [5]; “KIRA”) (Figure 1). GSK2850163 is a potent and selective dual inhibitor of the IRE1 kinase (IC_50_ = 20 nM) and RNase activities (IC_50_ = 200 nM) [4]. Despite its promising ability to modulate IRE1, this class of compounds was not developed further (undisclosed reasons from Glaxo SmithKline) [5]. The sulfonamide compound is an ATP‐competitive inhibitor based on an imidazopyrazine scaffold that inhibits RNase activity through the kinase domain of IRE1α i.e., kinase inhibiting RNase attenuator (KIRA). Several KIRA compounds have been proven useful to investigate the IRE1 activation mechanisms and the paralog‐specific function [3].

In more detail, we used these models to investigate the formation of the salt bridge interaction between (a) the β3‐strand lysine (Lys599 in IRE1) and the αC‐helix glutamate (Glu612 in IRE1), and (b) the β3‐strand lysine (Lys599 in IRE1) and aspartate (Asp711 in IRE1) of the DFG motif in the active and inactive conformations.

Our findings reveal a unique protonation‐dependent molecular switch that controls KIRA drug binding to the IRE1 kinase. Together these results provide novel insights on the prevalent view which assumes fixed protonation states i.e., that the β3‐strand catalytic lysine, the αC‐helix glutamate and the aspartate of the DFG motif in the active site are always charged (protonated/deprotonated), providing a new paradigm for the assessment of reactive lysine for designing targeted covalent kinase inhibitors [21]. Our study provides a solid foundation for further development of a thorough characterization of drug binding mechanisms in IRE1 and other kinases, that might lead to a novel structural paradigm in kinase drug discovery.

## 2 Materials and Methods

### 2.1 Protein Preparation

Structures of the cytosolic domain of human IRE1 in DICI conformation (PDB code 6W3B, active conformation [3]), DOCO conformation (PDB code 4YZ9, inactive conformation [4]) co‐crystallized with 2,7‐diazaspiro[4.5]decane inhibitor GSK2850163 [5], and DICO conformation (PDB code 4U6R, inactive conformation [6, 7]) co‐crystallized with a sulfonamide inhibitor [5] (Figure 1), were obtained from the RCSB Protein Data Bank. The structures were prepared using the Schrödinger protein preparation wizard [22]. The Prime program [23, 24] was employed to model absent sidechain atoms, and missing hydrogen atoms were added. The protonation states of ionizable residues were determined using the PROPKA tool [25] at pH = 7.4 followed by restrained minimizations (RMSD = 0.3 Å) using the OPLS4 force field [26].

### 2.2 QM/MM Calculations

The CP2K code [27, 28] was employed to carry out the QM/MM calculations on the cytosolic domain of IRE1. The systems were solvated in a cubic box with TIP3P [29] water molecules and 10 Å buffer distance. Afterwards, the systems were neutralized, and 0.15 M NaCl salt concentration added. The residues K599, E612, D711, and crystallized water plus ligand present in PDB codes: 4YZ9 (co‐crystallized with 2,7‐diazaspiro[4.5]decane inhibitor GSK285016314), and 4U6R (co‐crystallized with a sulfonamide inhibitor) were considered as the QM region and treated at the TPSS [30]‐DFTD3 [31]/DZVP‐MOLOPT‐GTH [32] level of density functional theory, whereas the rest of the cytosolic domain of IRE1 was treated as the MM region for which the AMBER14 force field (ff14SB) [33] was employed. The side chains of amino acid residues were cut through the Cα–Cβ bonds where Cα was a part of the MM while Cβ was a part of the QM subsystem. The linker atoms between QM and MM subsystems were treated using Integrated Molecular Orbital Molecular Mechanics (IMOMM) method [34]. The co‐crystallized ligands were prepared with LigPrep/Epik at pH 7.4 (stereochemistry fixed from the crystal). Thereafter, ligands were parameterized with GAFF as implemented in Ambertools2018 using the Antechamber interface tool [35]. The AM1‐BCC atomic point charges [36] were calculated using Antechamber [35, 37]. Finally, ligands were considered as the QM region and treated at the TPSS [30]‐DFTD3 [31]/DZVP‐MOLOPT‐GTH [32] level of density functional theory. Therefore, the QM region comprised residues Lys599, Glu612, Asp711, the co‐crystallized ligand (GSK2850163 in PDB 4YZ9; sulfonamide inhibitor KIRA in PDB 4U6R), and the active‐site water molecule (*W*_active site_, Figure S1). This water is structurally conserved in PDB 4YZ9 and 4U6R and was included to accurately model proton transfer dynamics and electrostatic interactions within the catalytic site.

Prior to the QM/MM energy optimization, the systems were equilibrated by conducting 5 ps NVT and 25 ps NPT classical MD simulation where the temperature and pressure were controlled at 298 K and 1 atm using the canonical sampling through velocity rescaling (CSVR) thermostat and barostat, respectively [38], with 100 fs relaxation time in both and a fixed time step of 0.5 fs. A 10 Å cut‐off distance was applied for non‐bonded interactions, while smooth particle‐mesh Ewald (SPME) summation [39] was used for the long‐range electrostatic interactions. No positional restraints or constraints were applied to any atoms‐protein, ligand, solvent, or ions‐during the NVT and NPT pre‐equilibration stages. All subsequent QM/MM geometry optimizations and WT‐MetaD simulations were performed without any positional restraints or constraints. The system was entirely free to explore the configurational space defined by the QM and MM potentials and the metadynamics bias. The structure optimization started with Lys599, Glu612, and Asp711 in their respective charged states.

### 2.3 QM/MM Metadynamics Simulations

To further investigate the formation/disruption of crucial hydrogen bonds in the triad Lys599–Glu612–Asp711 and confirm the unique protonation state of Lys599, the CP2K code [27, 28] version 8.2 patched with enhanced sampling library PLUMED 2.7.2 [40, 41] was employed to perform QM/MM well‐tempered metadynamics (WT‐MetaD) simulations. The PBE [42]‐DFTD3 [31]/DZVP‐MOLOPT‐GTH [32] level of density functional theory was employed for the QM region. The MM subsystem was modeled using the AMBER14 force field (ff14SB).

The collective variables (CVs) for the QM/MM WT‐MetaD simulations were carefully chosen to fully investigate salt bridge occurrence between the β3‐strand lysine (Lys599 in IRE1) and the αC‐helix glutamate (Glu612 in IRE1), and between the β3‐strand lysine (Lys599 in IRE1) and aspartate of the DFG motif (Asp711 in IRE1) in the active and inactive conformations.

As regards the apo state of IRE1 (PDB code: 6W3B, “apo” model), CV1 (Figure S2) accounts for the hydrogen bond between Lys599 and Glu612, defined as the difference between the NZ_Lys599_‐H_Lys599_ and H_Lys599_‐O_Glu612_ distances:

(1) $$\begin{array}{c} \text{CV1}=\text{𝑑}\left({\mathrm{NZ}}_{\mathrm{Lys}599}-{\text{HZ3}}_{\mathrm{Lys}599}\right)-\text{𝑑}\left({\text{HZ3}}_{\mathrm{Lys}599}-{\text{OE1}}_{\mathrm{Glu}612}\right)\\ \;\left(\text{upperwall}=+2.0,\text{lowerwall}=-2.0\right) \end{array}$$

CV2 (Figure S2) accounts for the hydrogen bond between Lys599 and Asp711 defined as the difference between the NZ_Lys599_‐H_Lys599_ and H_Lys599_‐O_Asp711_ distances:

(2) $$\begin{array}{c} \text{CV2}=\text{𝑑}\left({\mathrm{NZ}}_{\mathrm{Lys}599}-{\text{HZ2}}_{\mathrm{Lys}599}\right)-\text{𝑑}\left({\text{HZ2}}_{\mathrm{Lys}599}-{\text{OD1}}_{\mathrm{Asp}711}\right)\\ \;\left(\text{upperwall}=+2.0,\text{lowerwall}=-2.0\right) \end{array}$$

Regarding the inactive state of IRE1 co‐crystallized with the 2,7‐diazaspiro[4.5]decane inhibitor GSK2850163 (PDB code: 4YZ9, “GSK” model) [4] a single CV (Figure S3) accounting for the hydrogen bond between Lys599 and Asp711 defined as the difference between the NZ_Lys599_‐H_Lys599_ and H_Lys599_‐O_Asp711_ distances was employed:

(3) $$\begin{array}{c} \text{CV1}=\text{𝑑}\left({\mathrm{NZ}}_{\mathrm{Lys}599}-{\text{HZ2}}_{\mathrm{Lys}599}\right)-\text{𝑑}\left({\text{HZ2}}_{\mathrm{Lys}599}-{\text{OD2}}_{\mathrm{Asp}711}\right)\\ \;\left(\text{upperwall}=+2.0,\text{lowerwall}=-2.0\right) \end{array}$$

For the inactive state of IRE1 co‐crystallized with a sulfonamide inhibitor [5] (PDB code 4U6R [6, 7], “KIRA” model), a single CV (Figure S4) accounting for the hydrogen bond between Lys599 and Glu612, defined as the difference between NZ_Lys599_‐H_Lys599_ and H_Lys599_‐O_Glu612_ distances was used:

(4) $$\begin{array}{c} \text{CV1}=\text{𝑑}\left({\mathrm{NZ}}_{\mathrm{Lys}599}-{\text{HZ3}}_{\mathrm{Lys}599}\right)-\text{𝑑}\left({\text{HZ3}}_{\mathrm{Lys}599}-{\text{OE1}}_{\mathrm{Glu}612}\right)\\ \;\left(\text{upperwall}=+2.0,\text{lowerwall}=-2.0\right) \end{array}$$

To enhance the sampling procedure of the CV space, the system was biased by adding a gaussian kernel with the initial height of 0.5 kcal mol^−1^ and width of 0.25 Å every 100 MD steps (50 fs). A bias factor of 17.89 (i.e., 10 kcal mol^−1^) was set to scale down the height of spawning Gaussian kernels. The rest of the settings were the same as in the classical MD simulations. The QM/MM WT‐MetaD simulations stopped when the system repeatedly explored both the reactant and product states, and the free energy surface (FES) overlapped consistently as a function of the simulation time. All the Transition states (TS) examined in this work were further analyzed by isocommittor analyses [43], where the point of maximal energy within the proton transfer process was designated as the initial reference. The analyses were conducted on configurations/structures corresponding to metadynamics frames that reside in this vicinity. A total of 20 independent unbiased dynamical trajectories, initiated with random initial velocities, were executed for the selected configurations, ceasing once the system transitioned to either the reactants or products state. The hypothesized TS for each reaction pathway was defined as the configuration where the proton transfer ratio between Lys599/Glu612 (or Asp711) is approximating 50% (Figures S5–S10).

### 2.4 MD Simulations

Human IRE1 cytosolic structures in DICI conformation (PDB code 6W3B, active conformation [3]), DICO conformation (PDB code 4U6R, inactive conformation [6, 7]) co‐crystallized with a sulfonamide inhibitor [5] with Lys599 and Glu612 in the neutral state, and DOCO conformation (PDB code 4YZ9, inactive conformation [4]) co‐crystallized with 2,7‐diazaspiro[4.5]decane inhibitor GSK2850163 [5] were subjected to MD simulations in NPT ensemble employing the Desmond MD simulator engine in Schrödinger [44] with the OPLS4 force field [26].

The three systems were solvated with TIP3P water [45]. in cubic periodic boxes with a buffer distance of 10 Å to the walls. The systems were then neutralized, and additional Na^+^/Cl^−^ counter ions were added to reach a physiological salt concentration of 0.15 M. Temperature (300 K) and pressure (1 atm) were controlled using the Martyna–Tobias–Klein barostat [46] and the Nose–Hoover thermostat [47], respectively. The minimization and relaxation protocol consisted of (1) NVT Brownian dynamics with restraints on solute heavy atoms at *T* = 10 K for 100 ps, (2) a NVT simulation at *T* = 10 K with restraints on solute heavy atoms for 12 ps, (3) a NPT MD simulation at *T* = 10 K with restraints on solute heavy atoms for 12 ps, (4) a NPT MD simulation at *T* = 300 K with restraints on solute heavy atoms for 12 ps, and (5) a NPT MD simulation at *T* = 300 K without restraints for 24 ps. After equilibration, 500 ns MD simulations were carried out in triplicate. MD setup, including the number of counter‐ions and water molecules, and the time series of protein and ligand RMSD, are shown in Figures S11–S28.

## 3 Results and Discussion

### 3.1 QM/MM Calculations

In order to investigate the interaction network established between Lys599 and Glu612 / Asp711, a series of QM/MM calculations were conducted using TPSS‐DFT (see Methods for details). As starting structures for the DICI, DICO and DOCO systems, we employed the PDB structures 6W3B, 4U6R and 4YZ9, respectively. The residues Lys599, Glu612, and Asp711 were considered in their charged states according to the kinase paradigm assuming fixed solution protonation states—i.e., that the β3‐strand catalytic lysine is protonated, and the αC‐helix glutamate and the DFG motif aspartate are deprotonated [21]. All structures underwent structural and energy optimization calculations.

**FIGURE 2:**
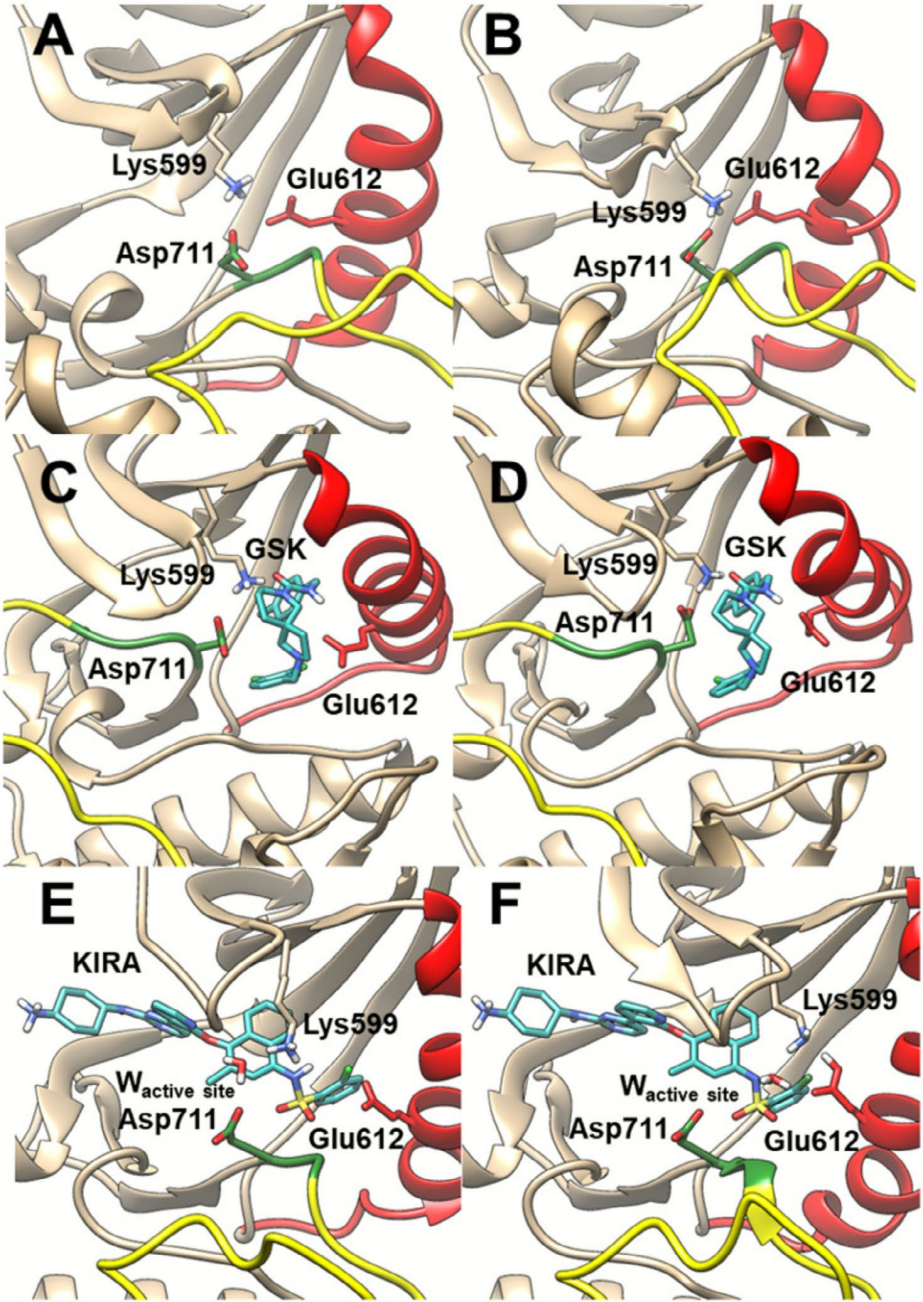
(A) Initial configuration of the apo active structure (PDB code: 6W3B), and (B) optimized configuration. (C) Initial configuration of the inactive conformation [4] co‐crystallized with GSK [5] (PDB code: 4YZ9), and (D) optimized configuration. (E) Initial configuration of the inactive conformation co‐crystallized with KIRA [5] (PDB code: 4U6R [6, 7]), and (F) optimized configuration.

For the apo state of IRE1 (PDB code: 6W3B, Figure 2A), upon structural and energy optimization *two charge‐enforced hydrogen bonds are formed by Lys599 with Glu612 and Asp711* (Figure 2B). The Lys599‐Asp711 interaction has N–H, H–O, and N–O distances 1.08, 1.62 and 2.70 Å, respectively, and N–H–O angle 174°, whereas the Lys599‐Glu612 interaction has N–H, H–O, and N–O distances 1.08, 1.63 and 2.67 Å, respectively, and N–H–O angle 158° (Figure 2B).

Regarding the DOCO system (“GSK”) [4], we found *only one single hydrogen bond between Lys599 and Asp711*, in agreement with the experimental evidence [5](Figure 2D). The hydrogen bond between the Lys599 and Asp711 of the optimized structure occurs with N–H, O–H and N–O distances of 1.09, 1.56, and 2.64 Å, respectively, and NHO angle 171°. It is worth noting that in the inactive conformation, the salt bridge between Glu612 and Lys599 is lost as shown by the distance between Lys599 and the αC‐helix Glu612 with OH, NH, and ON distances of 6.03, 1.06, and 6.77 Å, respectively (Figure 2D).

At variance with the previous two systems, during the energy optimization of the inactive KIRA bound system (DICO), *a proton transfer occurs from Lysine599 to Glu612*. This implies that Lys599 prefers to remain deprotonated inside the IRE1 kinase pocket in the presence of the sulfonamide inhibitor [5], and no interaction is formed between Lys599 and Asp711. The O–H, N–H, and O–N distances between Lys599 and Asp711 are 6.56, 1.03, and 7.57 Å, respectively (Figure 2F). This finding is in agreement with the experimental structure [7] where in this inactive conformation, no salt bridge between Lys599‐Asp711 is found. Interestingly, the water in the active site attracts the proton of the sulfonamide nitrogen i.e., KIRA loses its proton (forming SO_2_N^−^ + H_3_O^+^, Figure S29), a process that is not uncommon given the electron‐withdrawing character of the adjacent SO_2_ group and the aromatic scaffold [48]. The KIRA‐bound DICO system exhibits particularly interesting chemistry due to the acidic nature of the sulfonamide group. Sulfonamides (SO_2_NHR) are well‐known to be relatively acidic (pKa ~ 10–11 for simple aryl sulfonamides, but significantly lower for electron‐withdrawing substituents) [48].

### 3.2 QM/MM WT‐MetaD

In order to get a deeper insight into the energetic landscape of the formation/disruption of interactions in the triad Lys599‐Glu612‐Asp711 and the protonation transfer reaction occurring in the KIRA system, we performed QM/MM WT‐MetaD calculations on the apo, GSK, and KIRA systems using PBE [42]‐DFTD3 [31]/DZVP‐MOLOPT‐GTH [32] as the level of density functional theory for the QM part.

**FIGURE 3:**
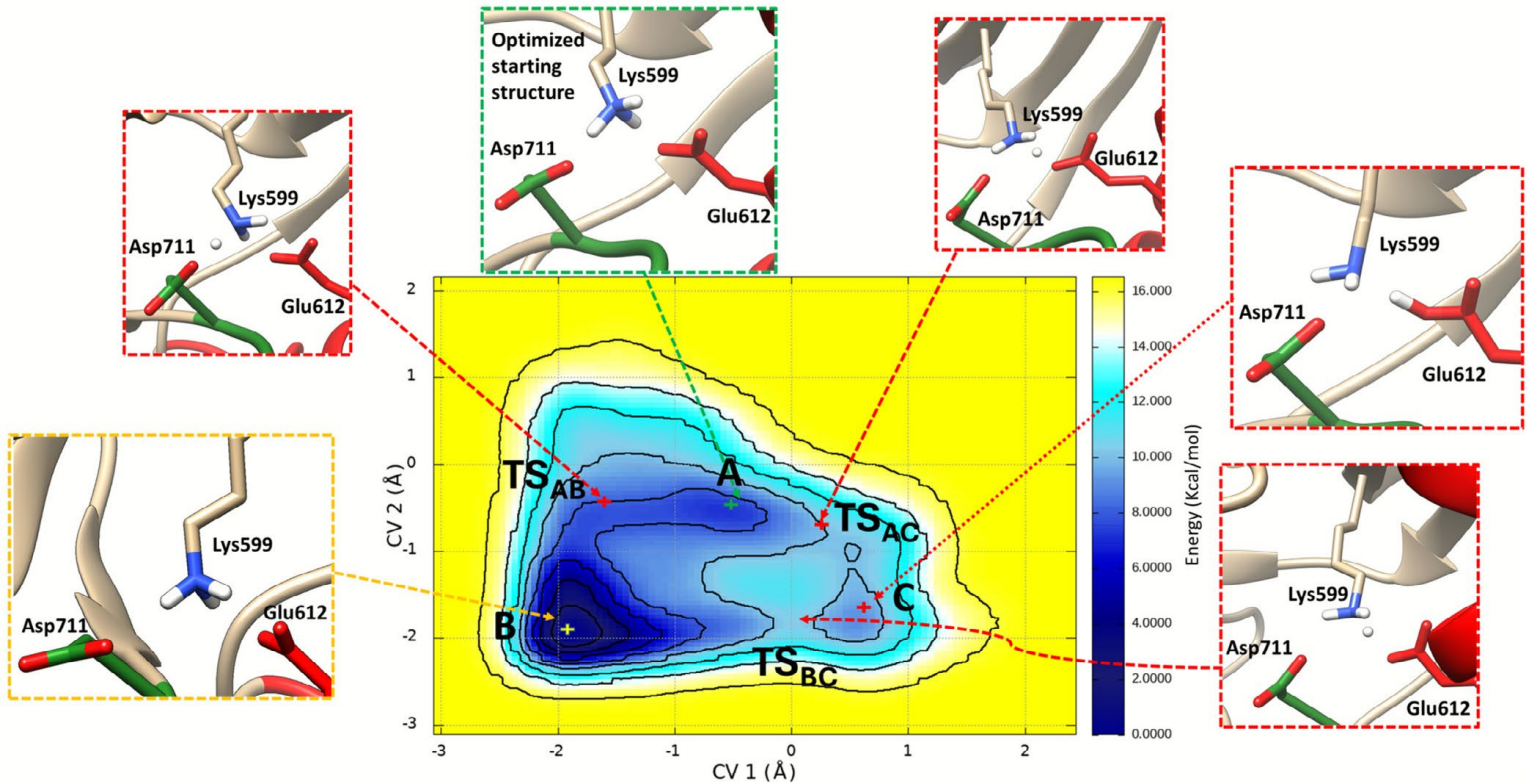
2D free energy surface (FES) as a function of CV1 and CV2 for the apo system. The inserts depict the three energetic minima: A = starting state, B = charged state (lowest energy state) and C = neutral state, respectively.

In Figure 3 we report the free energy surface (FES) computed for the IRE1 apo form as a function of the two collective variables (CVs) defining the charged states of Lys599, Glu612 and Asp711. CV1 accounts for the hydrogen bond between Lys599 and Glu612, defined as the difference between NZ_Lys599_‐H_Lys599_ and H_Lys599_‐O_Glu612_, while CV2 accounts for the hydrogen bond between Lys599 and Asp711, defined as the difference between NZ_Lys599_‐H_Lys599_ and H_Lys599_‐O_Asp711_. In the starting pose A, Lys599 forms hydrogen bonds with both Glu612 and Asp711, while the lowest energy state B is characterized by Lys599, Asp711, and Glu612 in their charged states. Interestingly, one additional energy minimum is found representing the state with both Lys599 and Glu612 in the neutral state (Point C), disclosing a unique (neutral) protonation state of Lys599. Indeed, previous *in vitro* experimental data [49, 50], mapped by high‐performance liquid chromatography (HPLC)‐linked mass spectrometry, revealed a peculiar reactive neutral Lys599 able to be decorated with hydroxyl aryl aldehyde‐like compounds via an imine bond, in accordance with the protonation state observed in our calculations. The transition state for proton transfer from Lys599 to Glu612 (TS_AC_, CV1 ≈ 0.3, CV2 ≈ −0.8) describes the proton as positioned midway between the Lys599 amine nitrogen (NZ) and the Glu612 carboxylate oxygen (OE1), with NZ–H and H–OE1 distances of 1.47 Å and 1.16 Å, respectively. Simultaneously, Lys599 maintains a hydrogen bond with Asp711 (NZ–H…OD1 distance: 1.78 Å), stabilizing the catalytic triad during the proton‐transfer event. The transition state lies above the starting pose A, representing the activation barrier for Lys599 deprotonation. The TS_AB_ for proton transfer from Lys599 to Asp711 (TS_AB_, CV1 ≈ −1.6 Å, CV2 ≈ −0.6 Å) shows analogous geometric features, with the proton shared between Lys599 and Asp711 (NZ–H: 1.30 Å; H–OD1: 1.38 Å). The transition state represents the activation barrier for starting pose A, where Lys599 forms hydrogen bonds with both Glu612 and Asp711, and the lowest energy state B characterized by Lys599, Asp711, and Glu612 in their charged states. TS_BC_ (CV1 ≈ 0.0 Å, CV2 ≈ −1.8 Å) describes the proton as positioned midway between the Lys599 amine nitrogen (NZ) and the Glu612 carboxylate oxygen (OE1), with NZ–H and H–OE1 distances of 1.13 Å and 1.45 Å, respectively. The transition state represents the activation barrier for Lys599 deprotonation toward Glu612.

**FIGURE 4:**
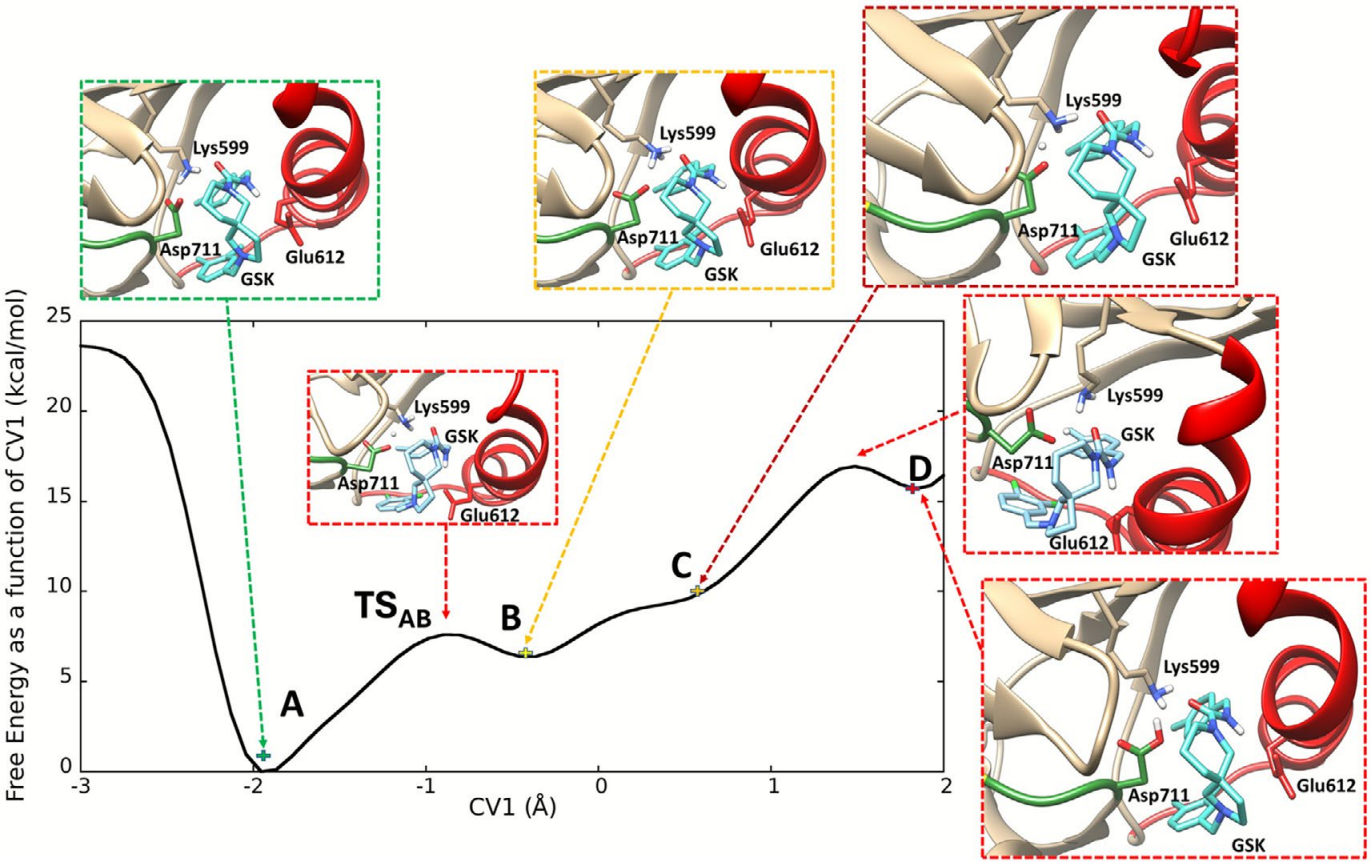
1D FES of the GSK system (PDB code:4YZ9). The three energetic minima A, B and D, and the inflection point for proton transfer (C), are shown as inserts.

Regarding the GSK system, the ligand occupies the space between Lys599 and Glu612, and we therefore computed the FES as a single CV, which defines the charged states of Lys599 and Asp711 (Figure 4), defined as the difference between the distances NZ_Lys599_‐H_Lys599_ and H_Lys599_‐O_Asp711_.

Three energy minima are found, the lowest (A in Figure 4) at CV = −1.95 Å representing the state with Lys599 and Asp711 in charged‐enforced hydrogen bonds, i.e., both oxygens of the carboxylate are involved in hydrogen bonding with Lys599, the second (B in Figure 4) ~7 kcal/mol above A, with Lys599 and Asp711 in charged states forming a single hydrogen bond, and the third (D in Figure 4) 15 kcal/mol higher in energy compared to A, with Lys599 and Asp711 in their neutral states.

As shown in Figure 4, moving from points B to D corresponds to the proton transfer between Lys599 and Asp711. This reaction requires passing through an “inflection point” point C, not representative of a minimum, therefore an unfavorable state. State C corresponds to a configuration in which Lys599 is transferring a proton to Asp711 (forming neutral Lys599 and protonated Asp711), but this state is thermodynamically unstable in the GSK‐bound system. The GSK ligand forms a stabilizing hydrogen bond with the protonated Lys599 (state B), which lowers the free energy of the charged Lys599‐Asp711 pair relative to the neutral pair. Committor analysis (Figure S9) confirms this interpretation: trajectories initiated from configurations near state C consistently relax back to state B (the charged Lys599‐Asp711 hydrogen‐bonded state) rather than proceeding forward to a stable neutral product. This indicates that state C is not a true free‐energy minimum but rather a transient, high‐energy configuration that the system escapes rapidly. However, we acknowledge the need for community‐wide validation efforts with smaller Gaussian heights for shallow barriers. Given the computational expense of QM/MM metadynamics (~1 ps/day), we selected 0.5 kcal/mol as a pragmatic compromise between exploration efficiency and accuracy. Still, this finding has important mechanistic implications: GSK binding stabilizes Lys599 in its charged (protonated) form, consistent with the experimental observation that GSK locks the kinase in the inactive DOCO conformation by disrupting the Lys599‐Glu612 salt bridge while maintaining Lys599‐Asp711 electrostatic interactions [51].

Furthermore, the ligand adopts a U‐shaped conformation disrupting the conserved H‐bond between the Glu612 and Lys599, thereby locking the αC‐helix in an inactive “out” conformation [5], with the protonated nitrogen of the pyrrolidine ring of the 2,8‐diazaspiro[4.5]decane forming a H‐bond with Glu612 and the oxygen of the carboxamide forming a H‐bond with Lys599. Indeed, in the GSK‐bound system, proton transfer occurs exclusively between Lys599 and Asp711, as the C‐helix (Glu612) is displaced by the GSK ligand and no longer in hydrogen‐bonding contact with Lys599. The transition state for proton transfer from Lys599 to Asp711 (TS_AB_, CV ≈ −0.9) describes the proton positioned midway between the Lys599 amine nitrogen (NZ) and the Asp711 carboxylate oxygen (OD2), with NZ–H and H–OD2 distances of 1.39 Å and 1.21 Å, respectively. The GSK ligand stabilizes this TS geometry by occupying the space between Lys599 and Glu612, thereby preventing their interaction and locking the C‐helix in the “out” conformation. This TS structure confirms that GSK binding disrupts the Lys599‐Glu612 salt bridge and redirects proton transfer exclusively to Asp711, consistent with the DOCO (DFG‐out, C‐helix‐out) inactive conformation observed experimentally.

**FIGURE 5:**
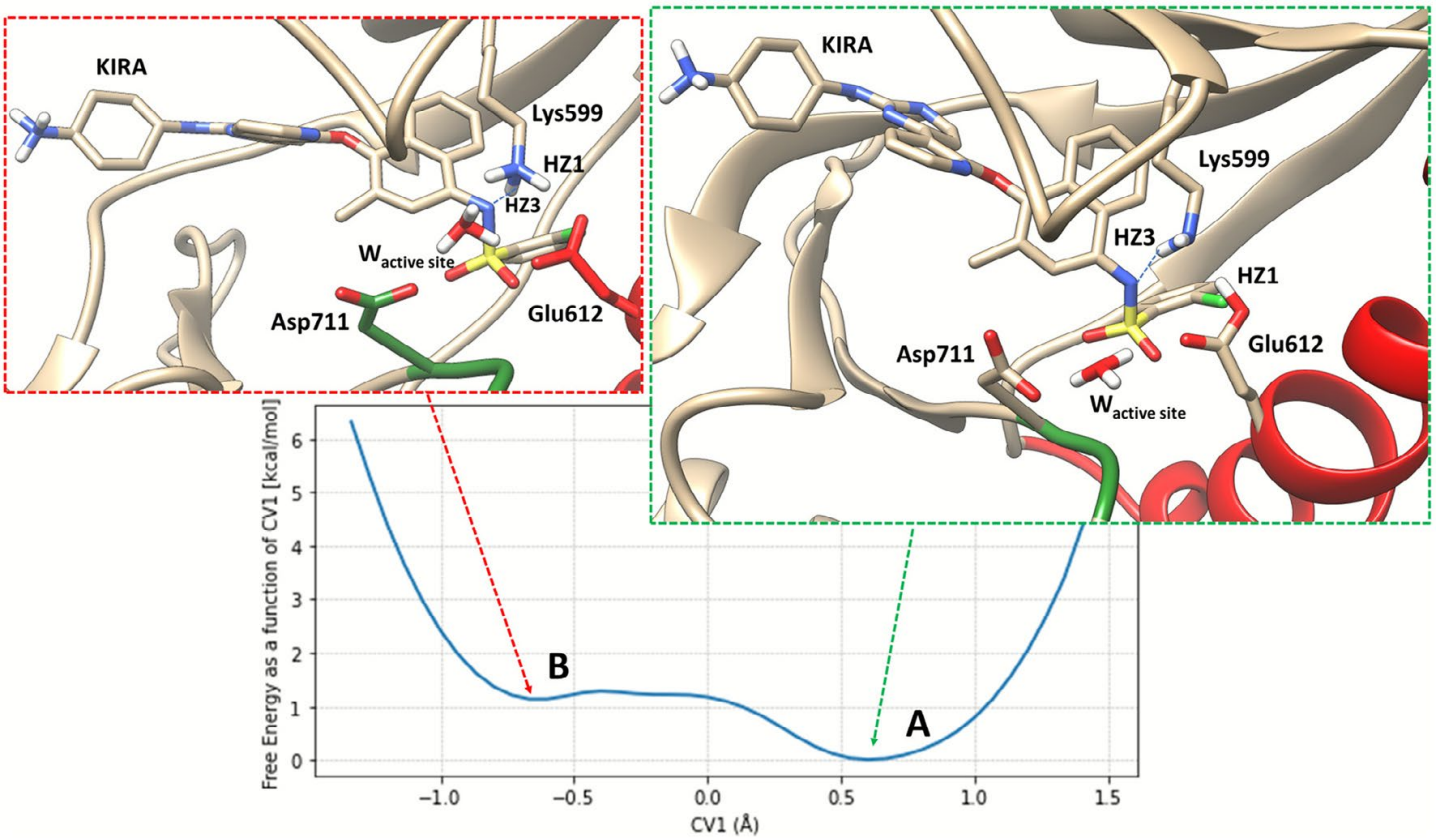
1D FES of the KIRA system (PDB code:4U6R). The local structure at the energetic minimum is shown as an insert.

When IRE1 is bound to the KIRA ligand, the FES is computed as a function of CV1 that defines the charged state of Lys599 and Glu612 (Figure 5), as the difference between the distances NZ_Lys599_‐H_Lys599_ and H_Lys599_‐O_Glu612_. The KIRA‐bound DICO system exhibits particularly interesting chemistry due to the acidic nature of the sulfonamide group. Sulfonamides (SO_2_NHR) are well‐known to be relatively acidic (pKa ~ 10–11 for simple aryl sulfonamides, but significantly lower for electron‐withdrawing substituents) [48]. In the KIRA ligand, the sulfonamide nitrogen can lose its proton (forming SO_2_N^−^ + H_3_O^+^), a process that is not uncommon given the electron‐withdrawing character of the adjacent SO_2_ group and the aromatic scaffold. Upon deprotonation, the nitrogen atom adopts a trigonal pyramidal (sp^2^) geometry, indicating that the lone pair resides on the nitrogen and is not stabilized in an imine‐like resonance structure [48]. When KIRA is deprotonated, the anionic nitrogen (SO_2_N^−^) becomes a strong hydrogen‐bond acceptor. Our QM/MM WT‐MetaD simulations reveal that HZ3 of Lys599 forms a persistent hydrogen bond with the deprotonated sulfonamide nitrogen throughout nearly the entire simulation trajectory. This interaction stabilizes the deprotonated KIRA state and perturbs the protonation equilibrium of Lys599. The deprotonation of KIRA fundamentally alters the electrostatic landscape of the active site, resulting in the unusual free‐energy surface (FES) sampled in Figure S30. The original CV [CV1 = d(NZ–HZ3) – d(HZ3–OE1_Glu612_)] was designed to capture proton transfer between Lys599 and Glu612. However, because HZ3 is engaged in hydrogen bonding with the deprotonated KIRA nitrogen rather than participating in proton transfer to Glu612, the biased CV does not accurately represent the true reaction coordinate for the Lys599‐Glu612 proton‐transfer event. To address this, we performed FES reweighting. Specifically, we projected the accumulated bias potential onto an alternative collective variable: CV1′ = d(NZ–HZ1) – d(HZ1–OE1_Glu612_), where HZ1 is the proton that actually interacts with the Glu612 carboxylate (Figure S31) and is free from the KIRA nitrogen interaction. By projecting the bias onto CV1′ (the NZ–HZ1–OE1 coordinate), we obtained a corrected FES that accurately represents the thermodynamics of the Lys599‐Glu612 proton‐transfer reaction in the presence of deprotonated KIRA (Figure 5, reweighted FES). The reweighted FES reveals that in State A, Lys599 has transferred the HZ1 proton to Glu612, resulting in neutral Lys599 and neutral (protonated) Glu612. In state B Lys599 is protonated, with HZ1 forming a hydrogen bond with Glu612 (OE1). The deprotonated KIRA nitrogen accepts a hydrogen bond from HZ3 and the active site water forms h‐bond with Asp711. The deprotonation of KIRA's sulfonamide group and its subsequent interaction with Lys599 (via HZ3) represent a ligand‐induced protonation‐state modulation that stabilizes the neutral form of Lys599 [49, 50, 52].

We note that the QMMM WT‐MetaD calculations are computationally costly with poor scalability (less than 1 ps/day). However, all the systems reached convergence as confirmed by the forth‐and‐back events observed between the low energy minima and the FES calculated as a function of the simulation time. The free‐energy calculations in the apo state of IRE1, the GSK system, and the KIRA system converged after ~25, ~25, and ~17 ps of QMMM WT‐MetaD calculations, respectively (Figures S32–S37).

Consistent with the protonation states of the triad Lys599‐Glu612‐Asp711 unraveled in this study, we were able to observe the different behavior of the αC‐helix during the MD simulations. Displacement of the αC‐helix occurred for the GSK and KIRA systems, as measured by the NZ_Lys599_‐C_Glu612_ distance during the MD trajectory (8.22 ± 1.20 and 12.38 ± 1.34 Å for KIRA and GSK, respectively) in contrast to the stable and favorable distance in the apo state of IRE1 (3.63 ± 0.30 Å). Indeed, the apo structure, i.e., the only one that assumes the active state in the DICI conformation, establishes conserved hydrogen bonds between Lys599‐Glu612 and Lys599‐Asp711 throughout the entire MD simulations (occupancy 77.8% and 75.6%, respectively). In contrast, the GSK binding breaks the hydrogen bond between Lys599‐Glu612 (0% throughout the entire MD simulations), locking the αC‐helix in an inactive “out” conformation while maintaining only one single hydrogen bond between Lys599 and Asp711 (72.4% over the simulations). Finally, in agreement with the experimental structure [5] KIRA disrupts all hydrogen bonds between the Lys599‐Glu612 pair and Asp711 (occupancy < 0.5% throughout the entire MD simulations).

Our results disclose a peculiar drug binding mechanism for IRE1 characterized by unique (neutral) protonation states of Lys599 and Glu612. Our study paves the way to further investigations on protonation‐dependent drug binding to the IRE1 kinase, for instance by performing high‐resolution neutron crystallographic analysis combined with high‐resolution X‐ray crystallography at room temperature [53, 54].

## 4 Conclusions

Herein, we investigated by means of quantum mechanical calculations, some key features of the ATP binding pocket of the IRE1 kinase domain, in its apo form and in complex with GSK and KIRA inhibitors. In agreement with the experimental structures, our simulations show the disruption of the Lys599‐Glu612 salt bridge in the inactive protein conformation [4] co‐crystallized with 2,7‐diazaspiro[4.5]decane inhibitor GSK2850163 [5] (PDB code: 4YZ9), and the inactive conformation co‐crystallized with a sulfonamide inhibitor [5](PDB code 4U6R [6, 7]). Interestingly, we found a unique protonation‐dependent molecular switch controlling KIRA drug binding to the IRE1 kinase by means of QM/MM calculations. The QM/MM calculations showed a spontaneous proton transfer from Lys599 to Glu612 in the KIRA complex. QMMM WT‐MetaD simulations provided structural and energetic details of this peculiar protonation‐dependent KIRA/IRE1 binding mode, which is similar to that found in ultrahigh‐resolution X‐ray crystal structures of CTX‐M β‐lactamase complex where the ligand induces a proton transfer from the catalytic Ser70 to Glu166 [55].

Our results indicate that the catalytic Lys599 in the IRE1 kinase domain may adopt a neutral state, i.e., function as a nucleophilic (reactive) catalytic lysine. As described in the constant pH molecular dynamics pKa prediction tool used for the identification of nucleophilic catalytic lysines and cysteines in various human kinase groups [21], the determination of nucleophilic catalytic lysines is significant for designing targeted covalent kinase inhibitors. Our finding is consistent with *in vitro* experimental data [49, 50], mapped by high‐performance liquid chromatography (HPLC)‐linked mass spectrometry, revealing an unusually reactive, i.e., neutral Lysine 599 able to react with hydroxyl aryl aldehyde like compounds via imine bond formation, which represents an unprecedented structural paradigm that could be employed in the rational design of new IRE1 inhibitors. Indeed, by identifying reactive catalytic lysines in human kinases, we can design novel lysine‐targeting covalent kinase inhibitors.

The proton transfer from Ly599 to Glu612 observed in the KIRA system disrupts a critical hydrogen bond that plays a key role in the IRE1 kinase's catalytic activity, through the displacement of the αC‐helix, stabilizing the inactive DICO conformation as observed experimentally [6, 7] and in MD simulations performed in this study. On the other hand, binding of the GSK (GSK system) completely breaks the salt bridge between Lys599‐Glu612 locking the αC‐helix in an inactive “out” conformation. At variance with the ligated forms of the kinase, the apo structure is the only one that can assume the active state in the DICI conformation where two salt bridges are formed by the triad Lys599‐Glu612‐Asp711.

Our findings are consistent with and extend the recent work by Liu et al. [56], who demonstrated that conformational transitions between active (DICI) and inactive (DOCO) kinase states are coupled to protonation‐state changes of the catalytic lysine. Using continuous constant pH molecular dynamics (CpHMD), they showed that the catalytic lysine adopts a reactive (neutral) state almost exclusively in the DOCO conformation, where it is not engaged in salt bridges with DFG‐Asp or αC‐Glu [56]. Our QM/MM metadynamics simulations provide complementary, atomistic insights into the proton‐transfer mechanisms underlying these protonation‐state changes. We find that:

1. In the apo DICI system, neutral Lys599‐Glu612 is a free‐energy minimum demonstrating that proton transfer from Lys599 to Glu612 is thermodynamically accessible even in the active conformation.
2. In the GSK‐bound DOCO system, however, neutral Lys599 is unstable (committor analysis confirms zero forward probability from the transient state C). This contrasts with the Tsai/Shen prediction that DOCO stabilizes neutral Lys, and highlights the critical role of ligand‐specific electrostatic interactions: GSK forms a stabilizing H‐bond with charged Lys599, thereby suppressing proton transfer to Asp711.
3. In the KIRA‐bound DICO system, neutral Lys599‐Glu612 becomes the global minimum, driven by the deprotonation of KIRA's sulfonamide group (SO_2_NH → SO_2_N^−^), which acts as a strong H‐bond acceptor for one of Lys599's hydrogens (HZ3).

Together, our results demonstrate that protonation states and conformational transitions are coupled, but the coupling is modulated by ligand identity as well. While the previous model provides a useful framework linking neutral Lys to the rare DOCO conformation, our study reveals that multiple inactive conformations (DOCO, DICO) can support neutral Lys, depending on the ligand‐induced electrostatic environment. This has important implications for rational inhibitor design: ligands can be designed to either stabilize neutral Lys (e.g., KIRA) for covalent targeting, or suppress neutral Lys (e.g., GSK) to lock the kinase in a specific inactive conformation.

Overall, our study reveals a novel structural paradigm in kinases in which ligand binding might alter the protonation state of Lys599 and Glu612, with important consequences on the reactivity of the enzyme and its druggability. For instance, one could design electrophilic compounds acting as strong stabilizers of the neutral form of Lys599, thus inducing a severe impediment for the enzyme reaction and its prolonged inhibition. Further studies are necessary to investigate if a similar mechanism might occur in diverse kinases and other proteins. Our simulation protocol might be employed to retrospectively and prospectively predict lysine reactivity in such systems, possibly with the experimental support of high‐resolution neutron and/or X‐ray crystallography [53, 54].

## Author Contributions

**Antonio Carlesso**, **Paolo Conflitti**, **Sayyed Jalil Mahdizadeh**, **Leif A. Eriksson**, and **Vittorio Limongelli:** conceptualization. **Antonio Carlesso**, **Paolo Conflitti**, **Sayyed Jalil Mahdizadeh**, **Giuseppe Deganutti**, and **Vittorio Limongelli:** formal analysis. **Antonio Carlesso**, **Leif A. Eriksson:** funding acquisition. **Antonio Carlesso**, **Paolo Conflitti**, **Sayyed Jalil Mahdizadeh**, **Giuseppe Deganutti**, and **Vittorio Limongelli:** investigation. **Antonio Carlesso**, **Paolo Conflitti**, and **Sayyed Jalil Mahdizadeh:** methodology. **Antonio Carlesso**, **Paolo Conflitti**, and **Sayyed Jalil Mahdizadeh:** writing – original draft. **Antonio Carlesso**, **Paolo Conflitti**, **Sayyed Jalil Mahdizadeh**, **Giuseppe Deganutti**, **Leif A. Eriksson**, and **Vittorio Limongelli:** writing – review and editing.

## Funding

This work was supported by Cancerfonden, 211447‐Pj, Elisabeth and Alfred Ahlqvist's Foundation, Postdoctoral funding, The Foundation Blanceflor, and H2020 European Research Council, 101001784.

## Disclosure

The supplementary figures illustrate the coordination dynamics and FES evolution of the Lys599‐Glu612 pair in apo and ligand‐bound IRE1 (with GSK and KIRA) during QM/MM WT‐MetaD simulations. They highlight key recrossing events and confirm FES convergence over simulation time, available as Supporting Information to the current publication, freely available at NNNNNN.

## Conflicts of Interest

The authors declare no conflicts of interest.

## Supporting information


**Data S1:** Supplementary figures.

## Data Availability

The data that support the findings of this study are openly available at zenodo.org, reference number 10.5281/zenodo.15424611.
