# Supplementary material for: QM/MM Free Energy Calculations of IRE1 Reveal a Unique Protonation State of the Catalytic Lys599
Source: J Comput Chem. 2025 Dec 4;46(31):e70288. doi: 10.1002/jcc.70288 (PMC12678879; doi:10.1002/jcc.70288)
Supplement: Supplementary file 1 — Data S1: Supplementary figures. [file JCC-46-0-s001.pdf]

# QM/MM free energy calculations of IRE1 reveal a unique protonation state of the catalytic Lys599

Antonio Carlesso[1], Paolo Conflitti[2], Sayyed Jalil Mahdizadeh[3], Giuseppe Deganutti[4], Leif A. Eriksson[*,3], and Vittorio Limongelli[*,2,5]

[1]*Department of Pharmacology, Sahlgrenska Academy, University of Gothenburg, Gothenburg, Sweden*

[2]*Università della Svizzera italiana (USI), Faculty of Biomedical Sciences, Euler Institute, Via G. Buffi 13, CH-6900 Lugano, Switzerland.*

[3]*Department of Chemistry and Molecular Biology, University of Gothenburg, 405 30 Göteborg, Sweden*

[4]*Centre for Health and Life Sciences, Coventry University, Coventry, UK*

[5]*Department of Pharmacy, University of Naples "Federico II", via D. Montesano 49, I-80131 Naples, Italy.*

[*]Corresponding author: Prof. Dr. Leif A. Eriksson, email: leif.eriksson@chem.gu.se

Prof. Dr. Vittorio Limongelli, email: vittoriolimongelli@gmail.com

## Supporting Information

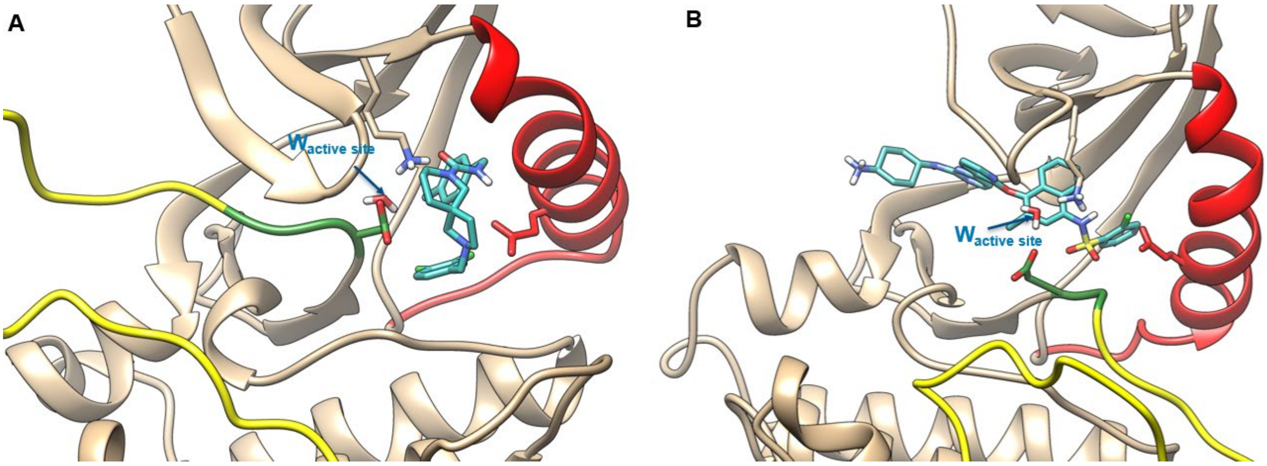


**Figure S1.** Location of the catalytic water molecule ($W_{cat}$) in the QM region. **(A)** Active site of IRE1 kinase domain from crystal structure (A) PDB 4YZ9 (DOCO conformation, GSK2850163-bound): the QM subsystem (shown in stick representation) includes residues Lys599, Glu612, Asp711, the co-crystallized ligand (GSK2850163), and the catalytic water molecule Wcat; **(B)** PDB 4U6R (DICO conformation, KIRA-bound). The QM subsystem includes residues Lys599, Glu612, Asp711, the co-crystallized ligand (KIRA6), and Wcat.

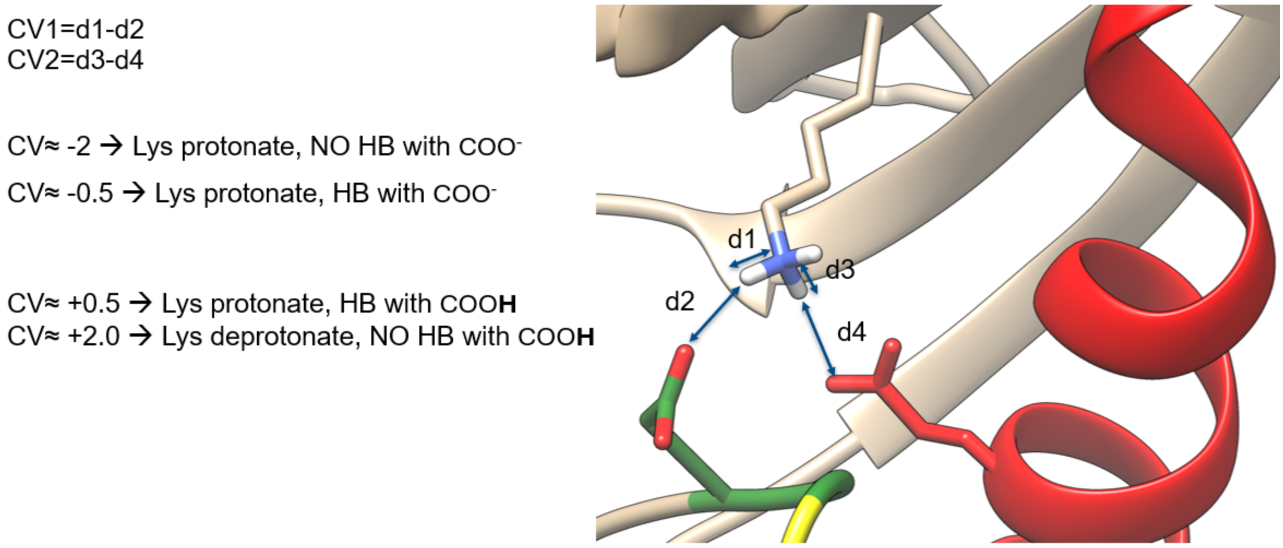


**Figure S2**. Schematic representation of the collective variables (CVs) used in QM/MM WT-MetaD simulation for apo state of IRE1 (PDB code: 6W3B). The panel depicts a close-up view of the active site, illustrating the atom selections and definitions used to construct the two collective variables: CV1 = d1 – d2, where d1 is the distance between the nitrogen atom of the Lys599 side-chain (NZ) and its own proton (HZ), and d2 is the distance between the same Lys599 proton (HZ) and the carboxylate oxygen (OE1) of Glu612. CV2 = d3 – d4, where d3 is the distance between Lys599 NZ–HZ and the carboxylate oxygen (OD1) of Asp711, and d4 is the distance between HZ and OD1 of Asp711. Sign convention: Negative CV values correspond to Lys protonated and hydrogen-bonded to Glu612 (or Asp711), while positive CV values correspond to Lys deprotonated and/or hydrogen-bonded to protonated Glu612 (COOH).

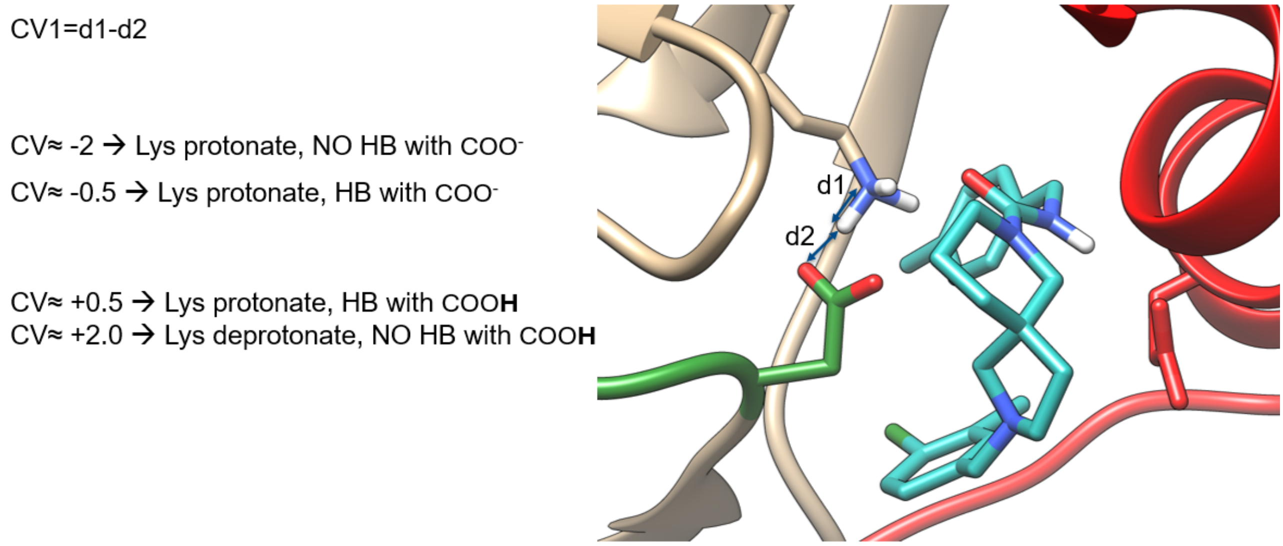


**Figure S3**. Schematic representation of the collective variables (CVs) used in QM/MM WT-MetaD simulation for GSK (PDB code: 4YZ9). The panel depicts a close-up view of the active site, illustrating the atom selections and definitions used to construct the two collective variables: CV1 = d1 – d2, where d1 is the distance between the nitrogen atom of the Lys599 side-chain (NZ) and its own proton (HZ), and d2 is the distance between the same Lys599 proton (HZ) and the carboxylate oxygen (OD2) of Asp711. Sign convention: Negative CV values correspond to Lys protonated and hydrogen-bonded to Asp711, while positive CV values correspond to Lys deprotonated and/or hydrogen-bonded to protonated Asp711 (COOH).

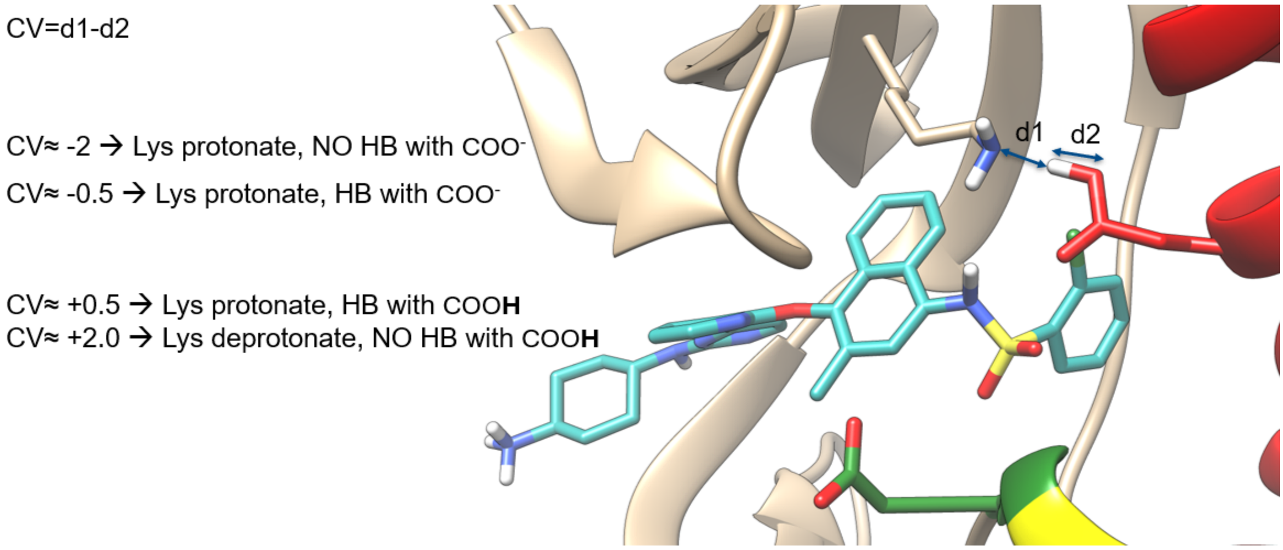


**Figure S4**. Schematic representation of the collective variables (CVs) used in QM/MM WT-MetaD simulation for KIRA (PDB code: 4U6R). The panel depicts a close-up view of the active site, illustrating the atom selections and definitions used to construct the two collective variables: CV1 = d1 – d2, where d1 is the distance between the nitrogen atom of the Lys599 side-chain (NZ) and its own proton (HZ), and d2 is the distance between the same Lys599 proton (HZ) and the carboxylate oxygen (OE1) of Glu612. Sign convention: Negative CV values correspond to Lys protonated and hydrogen-bonded to Glu612, while positive CV values correspond to Lys deprotonated and/or hydrogen-bonded to protonated Glu612 (COOH).

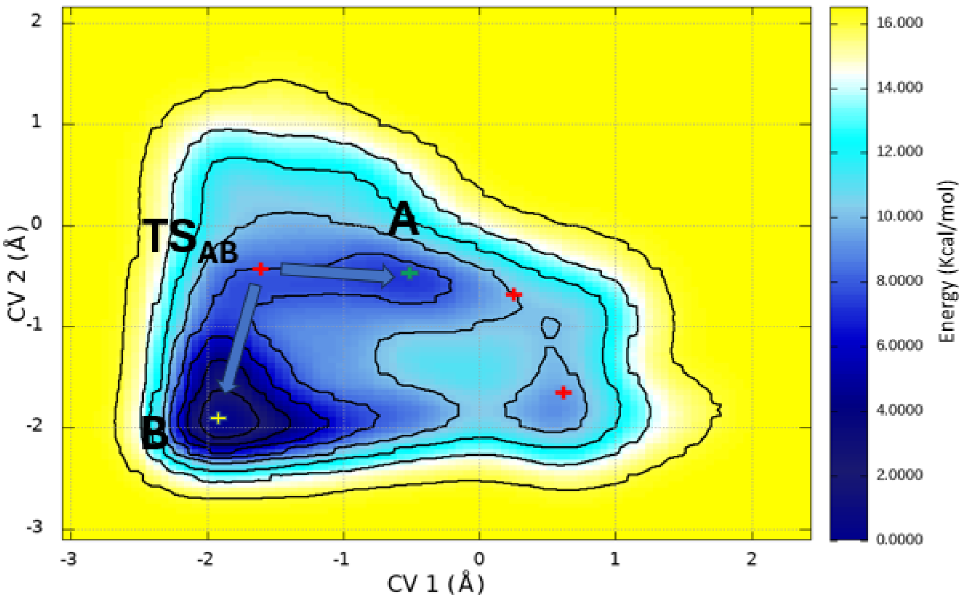


Replica 1 ---> B

```
#! FIELDS time diff1 diff2 basin
 4825.000000 -1.844631 -0.611590 1
#! FIELDS
#! SET COMMITTED TO BASIN 1
```

Replica 6 ---> A

```
#! FIELDS time diff1 diff2 basin
 4862.500000 -1.367664 -0.389732 2
#! FIELDS
#! SET COMMITTED TO BASIN 2
```

Replica 11 ---> A

```
#! FIELDS time diff1 diff2 basin
 4862.500000 -1.408622 -0.392394 2
#! FIELDS
#! SET COMMITTED TO BASIN 2
```

Replica 2 ---> B

```
#! FIELDS time diff1 diff2 basin
 4837.500000 -1.849292 -0.694921 1
#! FIELDS
#! SET COMMITTED TO BASIN 1
```

Replica 7 ---> B

```
#! FIELDS time diff1 diff2 basin
 4825.000000 -1.975473 -0.615129 1
#! FIELDS
#! SET COMMITTED TO BASIN 1
```

Replica 12 ---> A

```
#! FIELDS time diff1 diff2 basin
 4862.500000 -1.408622 -0.392394 2
#! FIELDS
#! SET COMMITTED TO BASIN 2
```

Replica 3 ---> A

```
#! FIELDS time diff1 diff2 basin
 4900.000000 -1.314521 -0.387928 2
#! FIELDS
#! SET COMMITTED TO BASIN 2
```

Replica 8 ---> B

```
#! FIELDS time diff1 diff2 basin
 4825.000000 -1.832432 -0.597559 1
#! FIELDS
#! SET COMMITTED TO BASIN 1
```

Replica 13 ---> B

```
#! FIELDS time diff1 diff2 basin
 4825.000000 -1.888584 -0.658114 1
#! FIELDS
#! SET COMMITTED TO BASIN 1
```

Replica 4 ---> B

```
#! FIELDS time diff1 diff2 basin
 4825.000000 -1.770014 -0.685587 1
#! FIELDS
#! SET COMMITTED TO BASIN 1
```

Replica 9 ---> A

```
#! FIELDS time diff1 diff2 basin
 4875.000000 -1.425055 -0.394093 2
#! FIELDS
#! SET COMMITTED TO BASIN 2
```

Replica 14 ---> B

```
#! FIELDS time diff1 diff2 basin
 4825.000000 -1.783976 -0.673643 1
#! FIELDS
#! SET COMMITTED TO BASIN 1
```

Replica 5 ---> A

```
#! FIELDS time diff1 diff2 basin
 4887.500000 -1.375562 -0.351170 2
#! FIELDS
#! SET COMMITTED TO BASIN 2
```

Replica 10 ---> B

```
#! FIELDS time diff1 diff2 basin
 4837.500000 -1.892198 -0.620709 1
#! FIELDS
#! SET COMMITTED TO BASIN 1
```

Replica 15 ---> B

```
#! FIELDS time diff1 diff2 basin
 4837.500000 -1.948248 -0.616267 1
#! FIELDS
#! SET COMMITTED TO BASIN 1
```

Replica 16 ---> B

```
#! FIELDS time diff1 diff2 basin
 4825.000000 -1.956198 -0.689647 1
#! FIELDS
#! SET COMMITTED TO BASIN 1
```

Replica 17 ---> B

```
#! FIELDS time diff1 diff2 basin
 4850.000000 -1.701207 -0.713747 1
#! FIELDS
#! SET COMMITTED TO BASIN 1
```

Replica 18 ---> A

```
#! FIELDS time diff1 diff2 basin
 4887.500000 -1.327868 -0.323725 2
#! FIELDS
#! SET COMMITTED TO BASIN 2
```

Replica 19 ---> A

```
#! FIELDS time diff1 diff2 basin
 4862.500000 -1.409959 -0.357375 2
#! FIELDS
#! SET COMMITTED TO BASIN 2
```

Replica 20 ---> A

```
#! FIELDS time diff1 diff2 basin
 4887.500000 -1.448020 -0.381437 2
#! FIELDS
#! SET COMMITTED TO BASIN 2
```

**Figure S5.** Isocommittor analysis of TS$_{AB}$ (apo, active conformation). From 20 independent simulations, ≈50% each of the trajectories converged to state A (9 simulations) or B (11 simulations).

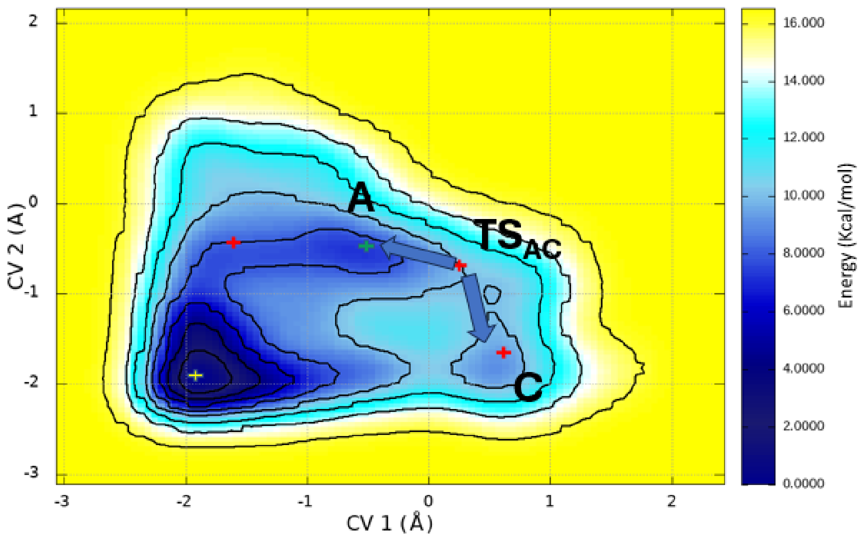


Replica 1 ---> A

```
#! FIELDS time diff1 diff2 basin
 10175.000000 -0.426557 -0.791029 1
#! FIELDS
#! SET COMMITTED TO BASIN 1
```

Replica 2 ---> C

```
#! FIELDS time diff1 diff2 basin
 10125.000000 0.687513 -0.981507 2
#! FIELDS
#! SET COMMITTED TO BASIN 2
```

Replica 3 ---> A

```
#! FIELDS time diff1 diff2 basin
 10212.500000 -0.293430 -0.681643 1
#! FIELDS
#! SET COMMITTED TO BASIN 1
```

Replica 4 ---> C

```
#! FIELDS time diff1 diff2 basin
 10112.500000 0.956819 -0.933873 2
#! FIELDS
#! SET COMMITTED TO BASIN 2
```

Replica 5 ---> A

```
#! FIELDS time diff1 diff2 basin
 10187.500000 -0.386328 -0.772595 1
#! FIELDS
#! SET COMMITTED TO BASIN 1
```

Replica 6 ---> A

```
#! FIELDS time diff1 diff2 basin
 10137.500000 0.133383 -0.772545 1
#! FIELDS
#! SET COMMITTED TO BASIN 1
```

Replica 7 ---> C

```
#! FIELDS time diff1 diff2 basin
 10112.500000 0.828700 -0.928757 2
#! FIELDS
#! SET COMMITTED TO BASIN 2
```

Replica 8 ---> C

```
#! FIELDS time diff1 diff2 basin
 10112.500000 0.828700 -0.928757 2
#! FIELDS
#! SET COMMITTED TO BASIN 2
```

Replica 9 ---> C

```
#! FIELDS time diff1 diff2 basin
 10125.000000 0.870779 -0.982545 2
#! FIELDS
#! SET COMMITTED TO BASIN 2
```

Replica 10 ---> A

```
#! FIELDS time diff1 diff2 basin
 10162.500000 0.167425 -0.758115 1
#! FIELDS
#! SET COMMITTED TO BASIN 1
```

Replica 11 ---> A

```
#! FIELDS time diff1 diff2 basin
 10187.500000 -0.610284 -0.643225 1
#! FIELDS
#! SET COMMITTED TO BASIN 1
```

Replica 12 ---> A

```
#! FIELDS time diff1 diff2 basin
 10175.000000 -0.094036 -0.705439 1
#! FIELDS
#! SET COMMITTED TO BASIN 1
```

Replica 13 ---> C

```
#! FIELDS time diff1 diff2 basin
 10125.000000 0.444412 -0.941408 2
#! FIELDS
#! SET COMMITTED TO BASIN 2
```

Replica 14 ---> C

```
#! FIELDS time diff1 diff2 basin
 10125.000000 0.805643 -0.911842 2
#! FIELDS
#! SET COMMITTED TO BASIN 2
```

Replica 15 ---> A

```
#! FIELDS time diff1 diff2 basin
 10175.000000 -0.094204 -0.715792 1
#! FIELDS
#! SET COMMITTED TO BASIN 1
```

Replica 16 ---> A

```
#! FIELDS time diff1 diff2 basin
 10187.500000 -0.568240 -0.746240 1
#! FIELDS
#! SET COMMITTED TO BASIN 1
```

Replica 17 ---> A

```
#! FIELDS time diff1 diff2 basin
 10137.500000 -0.014004 -0.695614 1
#! FIELDS
#! SET COMMITTED TO BASIN 1
```

Replica 18 ---> A

```
#! FIELDS time diff1 diff2 basin
 10162.500000 0.002020 -0.588124 1
#! FIELDS
#! SET COMMITTED TO BASIN 1
```

Replica 19 ---> C

```
#! FIELDS time diff1 diff2 basin
 10125.000000 0.495598 -0.985677 2
#! FIELDS
#! SET COMMITTED TO BASIN 2
```

Replica 20 ---> C

```
#! FIELDS time diff1 diff2 basin
 10125.000000 0.444412 -0.941408 2
#! FIELDS
#! SET COMMITTED TO BASIN 2
```

**Figure S6.** Isocommittor analysis of $TS_{AC}$ (apo, active conformation). From 20 independent simulations, ≈50% each of the trajectories converged to state A (11 simulations) or C (9 simulations).

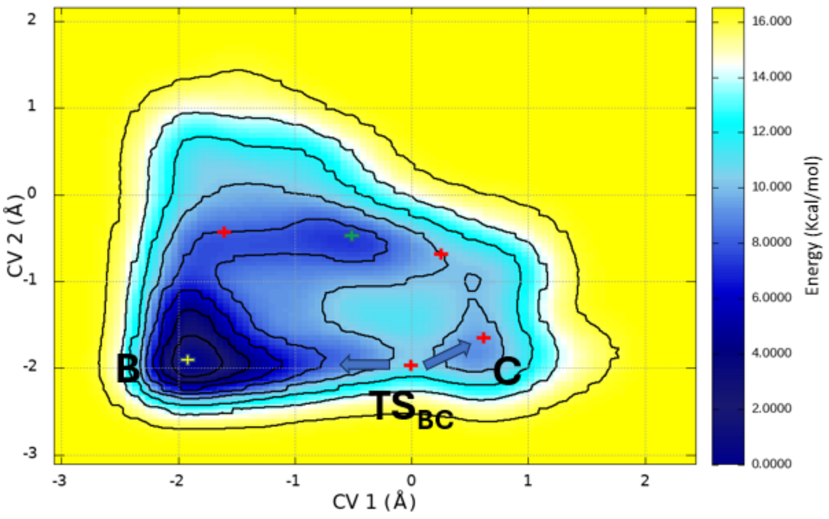


Replica 1 ---> C
```
#! FIELDS time diff1 basin
 9162.500000 0.639817 2
#! FIELDS
#! SET COMMITTED TO BASIN 2
```

Replica 2 ---> C
```
#! FIELDS time diff1 basin
 9162.500000 0.592912 2
#! FIELDS
#! SET COMMITTED TO BASIN 2
```

Replica 3 ---> B
```
#! FIELDS time diff1 basin
 2675.000000 -0.441521 1
#! FIELDS
#! SET COMMITTED TO BASIN 1
```

Replica 4 ---> C
```
#! FIELDS time diff1 basin
 9175.000000 0.595564 2
#! FIELDS
#! SET COMMITTED TO BASIN 2
```

Replica 5 ---> B
```
#! FIELDS time diff1 basin
 2687.500000 -0.341519 1
#! FIELDS
#! SET COMMITTED TO BASIN 1
```

Replica 6 ---> B
```
#! FIELDS time diff1 basin
 2662.500000 -0.380237 1
#! FIELDS
#! SET COMMITTED TO BASIN 1
```

Replica 7 ---> C
```
#! FIELDS time diff1 basin
 9162.500000 0.500656 2
#! FIELDS
#! SET COMMITTED TO BASIN 2
```

Replica 8 ---> C
```
#! FIELDS time diff1 basin
 9162.500000 0.442541 2
#! FIELDS
#! SET COMMITTED TO BASIN 2
```

Replica 9 ---> B
```
#! FIELDS time diff1 basin
 2675.000000 -0.631641 1
#! FIELDS
#! SET COMMITTED TO BASIN 1
```

Replica 10 ---> C
```
#! FIELDS time diff1 basin
 9162.500000 0.696842 2
#! FIELDS
#! SET COMMITTED TO BASIN 2
```

Replica 11 ---> B
```
#! FIELDS time diff1 basin
 2687.500000 -0.514363 1
#! FIELDS
#! SET COMMITTED TO BASIN 1
```

Replica 12 ---> C
```
#! FIELDS time diff1 basin
 9162.500000 0.285300 2
#! FIELDS
#! SET COMMITTED TO BASIN 2
```

Replica 13 ---> C
```
#! FIELDS time diff1 basin
 9162.500000 0.326445 2
#! FIELDS
#! SET COMMITTED TO BASIN 2
```

Replica 14 ---> B
```
#! FIELDS time diff1 basin
 2662.500000 -0.308954 1
#! FIELDS
#! SET COMMITTED TO BASIN 1
```

Replica 15 ---> C
```
#! FIELDS time diff1 basin
 9162.500000 0.479623 2
#! FIELDS
#! SET COMMITTED TO BASIN 2
```

Replica 16 ---> B
```
#! FIELDS time diff1 basin
 2675.000000 -0.417288 1
#! FIELDS
#! SET COMMITTED TO BASIN 1
```

Replica 17 ---> C
```
#! FIELDS time diff1 basin
 9162.500000 0.362436 2
#! FIELDS
#! SET COMMITTED TO BASIN 2
```

Replica 18 ---> C
```
#! FIELDS time diff1 basin
 9112.500000 0.692020 2
#! FIELDS
#! SET COMMITTED TO BASIN 2
```

Replica 19 ---> B
```
#! FIELDS time diff1 basin
 2675.000000 -0.465343 1
#! FIELDS
#! SET COMMITTED TO BASIN 1
```

Replica 20 ---> B
```
#! FIELDS time diff1 basin
 2687.500000 -0.479682 1
#! FIELDS
#! SET COMMITTED TO BASIN 1
```

**Figure S7.** Isocommittor analysis of $TS_{BC}$ (apo, active conformation). From 20 independent simulations, ≈50% each of the trajectories converged to the state B (9 simulations) or C (11 simulations).

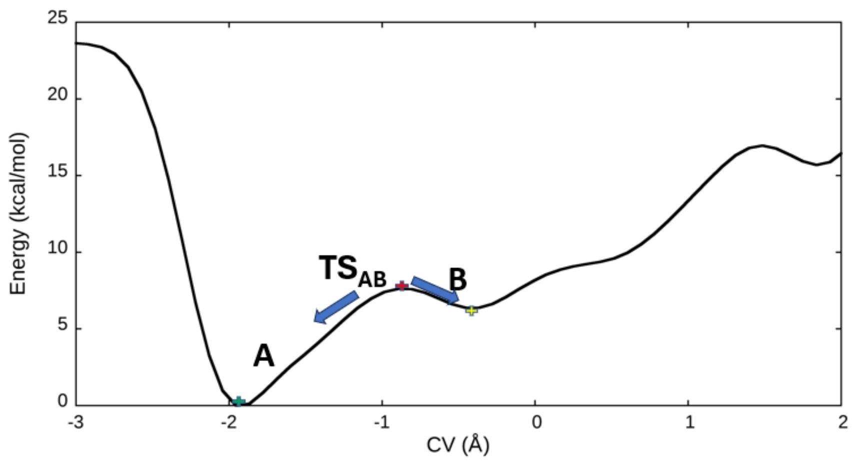


Replica 1 ---> B

```
#! FIELDS time diff1 basin
 14975.000000 -0.744731 2
#! FIELDS
#! SET COMMITTED TO BASIN 2
```

Replica 2 ---> A

```
#! FIELDS time diff1 basin
 14962.500000 -0.925695 1
#! FIELDS
#! SET COMMITTED TO BASIN 1
```

Replica 3 ---> A

```
#! FIELDS time diff1 basin
 14962.500000 -0.867802 1
#! FIELDS
#! SET COMMITTED TO BASIN 1
```

Replica 4 ---> B

```
#! FIELDS time diff1 basin
 14975.000000 -0.658476 2
#! FIELDS
#! SET COMMITTED TO BASIN 2
```

Replica 5 ---> B

```
#! FIELDS time diff1 basin
 14962.500000 -0.715960 2
#! FIELDS
#! SET COMMITTED TO BASIN 2
```

Replica 6 ---> B

```
#! FIELDS time diff1 basin
 14987.500000 -0.764087 2
#! FIELDS
#! SET COMMITTED TO BASIN 2
```

Replica 7 ---> B

```
#! FIELDS time diff1 basin
 14962.500000 -0.704110 2
#! FIELDS
#! SET COMMITTED TO BASIN 2
```

Replica 8 ---> A

```
#! FIELDS time diff1 basin
 14962.500000 -0.954659 1
#! FIELDS
#! SET COMMITTED TO BASIN 1
```

Replica 9 ---> A

```
#! FIELDS time diff1 basin
 14987.500000 -0.955479 1
#! FIELDS
#! SET COMMITTED TO BASIN 1
```

Replica 10 ---> A

```
#! FIELDS time diff1 basin
 14962.500000 -0.957496 1
#! FIELDS
#! SET COMMITTED TO BASIN 1
```

Replica 11 ---> B

```
#! FIELDS time diff1 basin
 14975.000000 -0.647803 2
#! FIELDS
#! SET COMMITTED TO BASIN 2
```

Replica 12 ---> B

```
#! FIELDS time diff1 basin
 14962.500000 -0.782010 2
#! FIELDS
#! SET COMMITTED TO BASIN 2
```

Replica 13 ---> B

```
#! FIELDS time diff1 basin
 14962.500000 -0.698469 2
#! FIELDS
#! SET COMMITTED TO BASIN 2
```

Replica 14 ---> A

```
#! FIELDS time diff1 basin
 14962.500000 -0.976125 1
#! FIELDS
#! SET COMMITTED TO BASIN 1
```

Replica 15 ---> B

```
#! FIELDS time diff1 basin
 14975.000000 -0.753999 2
#! FIELDS
#! SET COMMITTED TO BASIN 2
```

Replica 16 ---> A

```
#! FIELDS time diff1 basin
 14962.500000 -1.008479 1
#! FIELDS
#! SET COMMITTED TO BASIN 1
```

Replica 17 ---> A

```
#! FIELDS time diff1 basin
 14962.500000 -0.900455 1
#! FIELDS
#! SET COMMITTED TO BASIN 1
```

Replica 18 ---> B

```
#! FIELDS time diff1 basin
 14975.000000 -0.708532 2
#! FIELDS
#! SET COMMITTED TO BASIN 2
```

Replica 19 ---> B

```
#! FIELDS time diff1 basin
 14962.500000 -0.663310 2
#! FIELDS
#! SET COMMITTED TO BASIN 2
```

Replica 20 ---> B

```
#! FIELDS time diff1 basin
 14962.500000 -0.696042 2
#! FIELDS
#! SET COMMITTED TO BASIN 2
```

**Figure S8.** Isocommittor analysis of TS$_{AB}$ (GSK). From 20 independent simulations, ≈50% each of the trajectories converged to state A (8 simulations) or B (12 simulations).

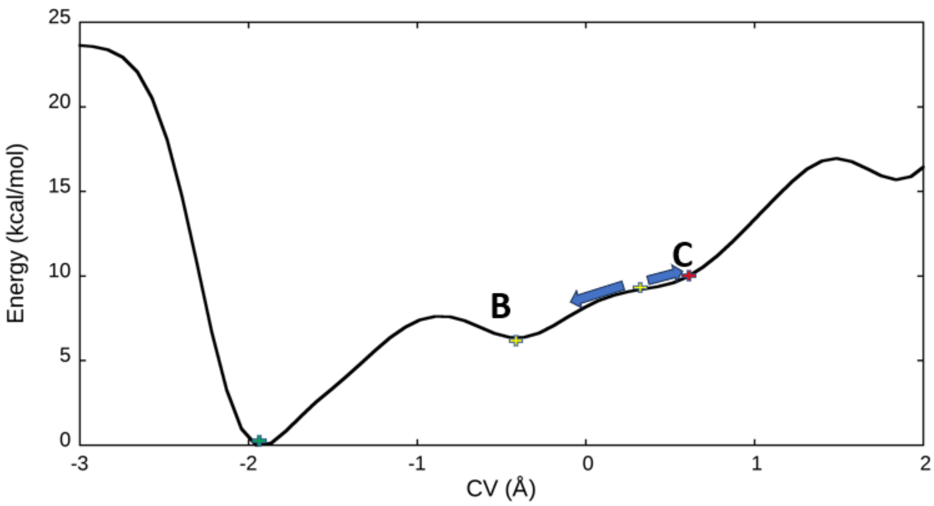


Replica 1 ---> B

```
#! FIELDS time diff1 basin
 6412.500000 -0.103262 1
#! FIELDS
#! SET COMMITTED TO BASIN 1
```

Replica 2 ---> B

```
#! FIELDS time diff1 basin
 6412.500000 0.314527 1
#! FIELDS
#! SET COMMITTED TO BASIN 1
```

Replica 3 ---> B

```
#! FIELDS time diff1 basin
 6412.500000 -0.203848 1
#! FIELDS
#! SET COMMITTED TO BASIN 1
```

Replica 4 ---> B

```
#! FIELDS time diff1 basin
 6412.500000 0.174631 1
#! FIELDS
#! SET COMMITTED TO BASIN 1
```

Replica 5 ---> B

```
#! FIELDS time diff1 basin
 6412.500000 0.253691 1
#! FIELDS
#! SET COMMITTED TO BASIN 1
```

Replica 6 ---> B

```
#! FIELDS time diff1 basin
 6412.500000 0.318737 1
#! FIELDS
#! SET COMMITTED TO BASIN 1
```

Replica 7 ---> B

```
#! FIELDS time diff1 basin
 6412.500000 -0.103262 1
#! FIELDS
#! SET COMMITTED TO BASIN 1
```

Replica 8 ---> B

```
#! FIELDS time diff1 basin
 6412.500000 0.042612 1
#! FIELDS
#! SET COMMITTED TO BASIN 1
```

Replica 9 ---> B

```
#! FIELDS time diff1 basin
 6412.500000 0.112454 1
#! FIELDS
#! SET COMMITTED TO BASIN 1
```

Replica 10 ---> B

```
#! FIELDS time diff1 basin
 6412.500000 0.207507 1
#! FIELDS
#! SET COMMITTED TO BASIN 1
```

Replica 11 ---> B

```
#! FIELDS time diff1 basin
 6412.500000 -0.077876 1
#! FIELDS
#! SET COMMITTED TO BASIN 1
```

Replica 12 ---> B

```
#! FIELDS time diff1 basin
 6412.500000 -0.065363 1
#! FIELDS
#! SET COMMITTED TO BASIN 1
```

Replica 13 ---> B

```
#! FIELDS time diff1 basin
 6412.500000 0.077641 1
#! FIELDS
#! SET COMMITTED TO BASIN 1
```

Replica 14 ---> B

```
#! FIELDS time diff1 basin
 6412.500000 0.062895 1
#! FIELDS
#! SET COMMITTED TO BASIN 1
```

Replica 15 ---> B

```
#! FIELDS time diff1 basin
 6412.500000 -0.493188 1
#! FIELDS
#! SET COMMITTED TO BASIN 1
```

Replica 16 ---> B

```
#! FIELDS time diff1 basin
 6412.500000 -0.099564 1
#! FIELDS
#! SET COMMITTED TO BASIN 1
```

Replica 17 ---> B

```
#! FIELDS time diff1 basin
 6425.000000 0.312256 1
#! FIELDS
#! SET COMMITTED TO BASIN 1
```

Replica 18 ---> B

```
#! FIELDS time diff1 basin
 6412.500000 -0.019533 1
#! FIELDS
#! SET COMMITTED TO BASIN 1
```

Replica 19 ---> B

```
#! FIELDS time diff1 basin
 6412.500000 -0.308374 1
#! FIELDS
#! SET COMMITTED TO BASIN 1
```

Replica 20 ---> B

```
#! FIELDS time diff1 basin
 6412.500000 -0.422363 1
#! FIELDS
#! SET COMMITTED TO BASIN 1
```

**Figure S9.** Isocommittor analysis of $TS_{BC}$ (GSK). From 20 independent simulations, 100% of the trajectories converged to state B.

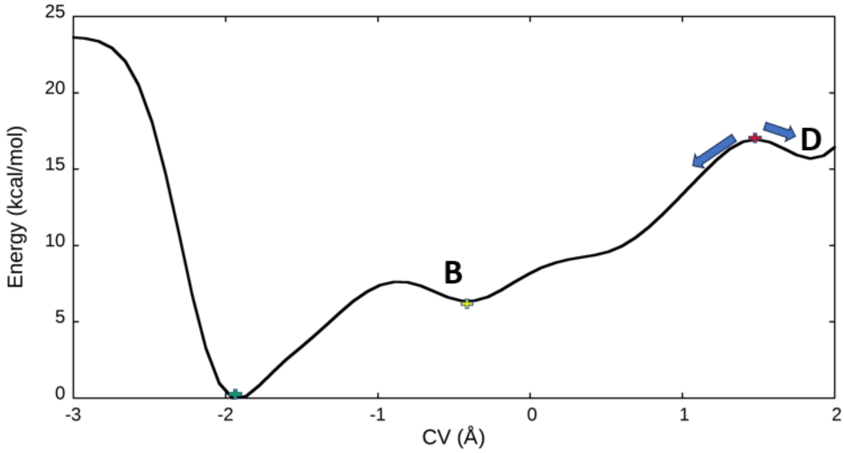


Replica 1 ---> B

```
#! FIELDS time diff1 basin
 10262.500000 1.228054 1
#! FIELDS
#! SET COMMITTED TO BASIN 1
```

Replica 2 ---> B

```
#! FIELDS time diff1 basin
 10262.500000 1.300357 1
#! FIELDS
#! SET COMMITTED TO BASIN 1
```

Replica 3 ---> D

```
#! FIELDS time diff1 basin
 10262.500000 1.888318 2
#! FIELDS
#! SET COMMITTED TO BASIN 2
```

Replica 4 ---> D

```
#! FIELDS time diff1 basin
 10275.000000 1.958161 2
#! FIELDS
#! SET COMMITTED TO BASIN 2
```

Replica 5 ---> D

```
#! FIELDS time diff1 basin
 10262.500000 1.544027 2
#! FIELDS
#! SET COMMITTED TO BASIN 2
```

Replica 6 ---> B

```
#! FIELDS time diff1 basin
 10262.500000 1.363681 1
#! FIELDS
#! SET COMMITTED TO BASIN 1
```

Replica 7 ---> D

```
#! FIELDS time diff1 basin
 10275.000000 1.628576 2
#! FIELDS
#! SET COMMITTED TO BASIN 2
```

Replica 8 ---> B

```
#! FIELDS time diff1 basin
 10262.500000 1.523903 2
#! FIELDS
#! SET COMMITTED TO BASIN 2
```

Replica 9 ---> D

```
#! FIELDS time diff1 basin
 10262.500000 1.665463 2
#! FIELDS
#! SET COMMITTED TO BASIN 2
```

Replica 10 ---> D

```
#! FIELDS time diff1 basin
 10262.500000 1.808180 2
#! FIELDS
#! SET COMMITTED TO BASIN 2
```

Replica 11 ---> B

```
#! FIELDS time diff1 basin
 10262.500000 1.377761 1
#! FIELDS
#! SET COMMITTED TO BASIN 1
```

Replica 12 ---> B

```
#! FIELDS time diff1 basin
 10262.500000 1.263269 1
#! FIELDS
#! SET COMMITTED TO BASIN 1
```

Replica 13 ---> D

```
#! FIELDS time diff1 basin
 10262.500000 1.305945 1
#! FIELDS
#! SET COMMITTED TO BASIN 1
```

Replica 14 ---> D

```
#! FIELDS time diff1 basin
 10262.500000 1.542791 2
#! FIELDS
#! SET COMMITTED TO BASIN 2
```

Replica 15 ---> D

```
#! FIELDS time diff1 basin
 10262.500000 1.798355 2
#! FIELDS
#! SET COMMITTED TO BASIN 2
```

Replica 16 ---> D

```
#! FIELDS time diff1 basin
 10262.500000 1.504188 2
#! FIELDS
#! SET COMMITTED TO BASIN 2
```

Replica 17 ---> D

```
#! FIELDS time diff1 basin
 10262.500000 1.903668 2
#! FIELDS
#! SET COMMITTED TO BASIN 2
```

Replica 18 ---> B

```
#! FIELDS time diff1 basin
 10262.500000 1.369612 1
#! FIELDS
#! SET COMMITTED TO BASIN 1
```

Replica 19 ---> D

```
#! FIELDS time diff1 basin
 10262.500000 1.543242 2
#! FIELDS
#! SET COMMITTED TO BASIN 2
```

Replica 20 ---> B

```
#! FIELDS time diff1 basin
 10262.500000 1.382114 1
#! FIELDS
#! SET COMMITTED TO BASIN 1
```

**Figure S10.** Isocommittor analysis of $TS_{BD}$ (GSK). From 20 independent simulations, ≈50% each of the trajectories converged to state B (8 simulations) or D (12 simulations).

| CPU # | Job Type | Ensemble | Temp. [K] | Sim. Time [ns] | # Atoms | # Waters | Charge |
|---|---|---|---|---|---|---|---|
| Unknown* | Unknown* | Unknown* | 300.0 | 501.001 | 127004 | 40126 | 0 |

* The configuration file (-out.cfg) was not found. Keep it in same directory as .aef file.

## Protein Information

| Tot. Residues | Prot. Chain(s) | Res. in Chain(s) | # Atoms | # Heavy Atoms | Charge |
|---|---|---|---|---|---|
| 398 | 'A' | ict_values([398] | 6401 | 3210 | -1 |

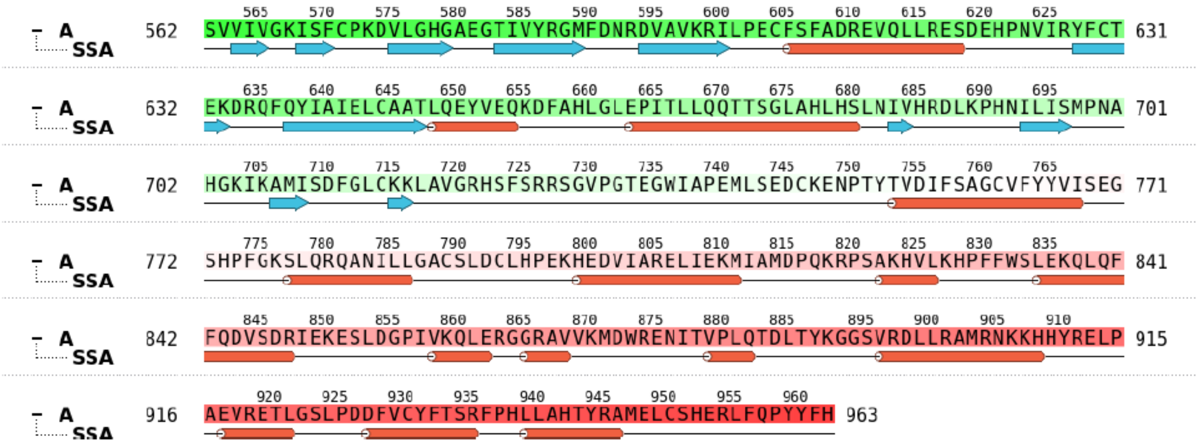


## Counter Ion/Salt Information

| Type | Num. | Concentration [mM] | Total Charge |
|---|---|---|---|
| Na | 113 | 51.202 | +113 |
| Cl | 112 | 50.749 | -112 |

**Figure S11.** MD setup for apo structure, replica 1.

| CPU # | Job Type | Ensemble | Temp. [K] | Sim. Time [ns] | # Atoms | # Waters | Charge |
|---|---|---|---|---|---|---|---|
| Unknown* | FEP | Unknown* | 300.0 | 501.004 | 127004 | 40126 | 0 |

* The configuration file (-out.cfg) was not found. Keep it in same directory as .aef file.

## Protein Information

| Tot. Residues | Prot. Chain(s) | Res. in Chain(s) | # Atoms | # Heavy Atoms | Charge |
|---|---|---|---|---|---|
| 398 | 'A' | ict_values([398] | 6401 | 3210 | -1 |

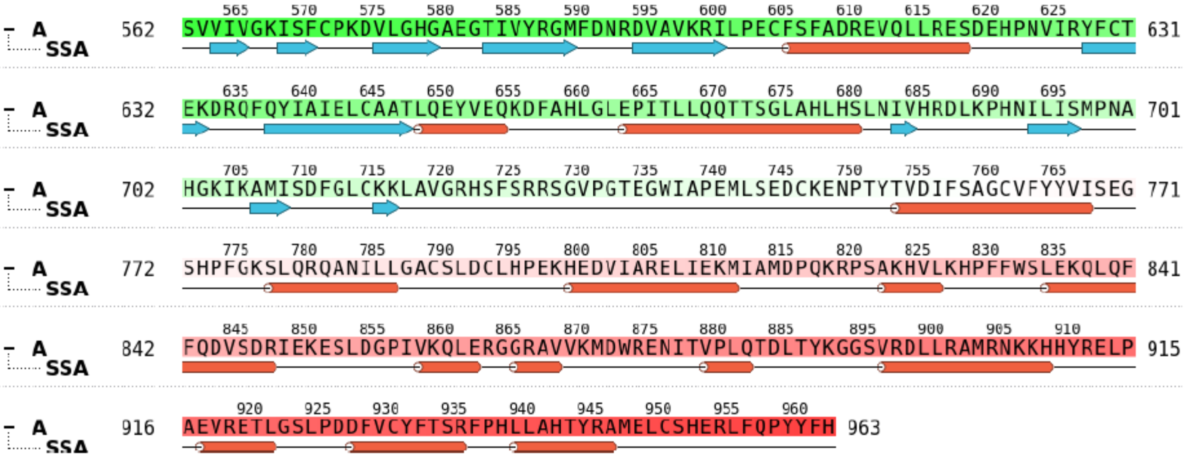


## Counter Ion/Salt Information

| Type | Num. | Concentration [mM] | Total Charge |
|---|---|---|---|
| Na | 113 | 51.202 | +113 |
| Cl | 112 | 50.749 | -112 |

**Figure S12.** MD setup for apo structure, replica 2.

| CPU # | Job Type | Ensemble | Temp. [K] | Sim. Time [ns] | # Atoms | # Waters | Charge |
|---|---|---|---|---|---|---|---|
| Unknown* | FEP | Unknown* | 300.0 | 500.504 | 127004 | 40126 | 0 |

* The configuration file (-out.cfg) was not found. Keep it in same directory as .aef file.

## Protein Information

| Tot. Residues | Prot. Chain(s) | Res. in Chain(s) | # Atoms | # Heavy Atoms | Charge |
|---|---|---|---|---|---|
| 398 | 'A' | ict_values([398] | 6401 | 3210 | -1 |

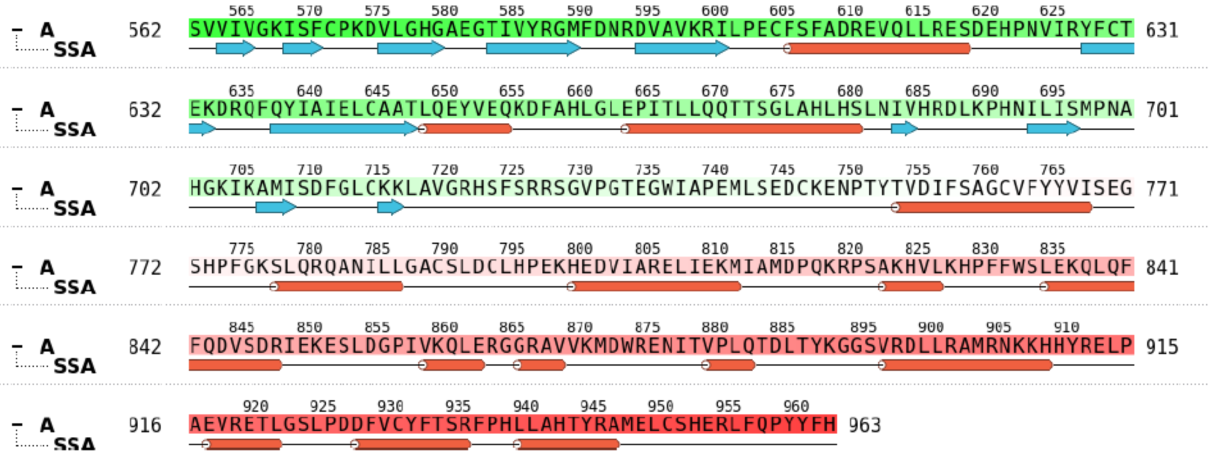


## Counter Ion/Salt Information

| Type | Num. | Concentration [mM] | Total Charge |
|---|---|---|---|
| Na | 113 | 51.202 | +113 |
| Cl | 112 | 50.749 | -112 |

**Figure S13.** MD setup for apo structure, replica 3.

| CPU # | Job Type | Ensemble | Temp. [K] | Sim. Time [ns] | # Atoms | # Waters | Charge |
|---|---|---|---|---|---|---|---|
| Unknown* | Unknown* | Unknown* | 300.0 | 501.001 | 149430 | 47695 | 0 |

* The configuration file (-out.cfg) was not found. Keep it in same directory as .aef file.

## Protein Information

| Tot. Residues | Prot. Chain(s) | Res. in Chain(s) | # Atoms | # Heavy Atoms | Charge |
|---|---|---|---|---|---|
| 374 | 'A' | ict_values([374] | 6019 | 3018 | -1 |

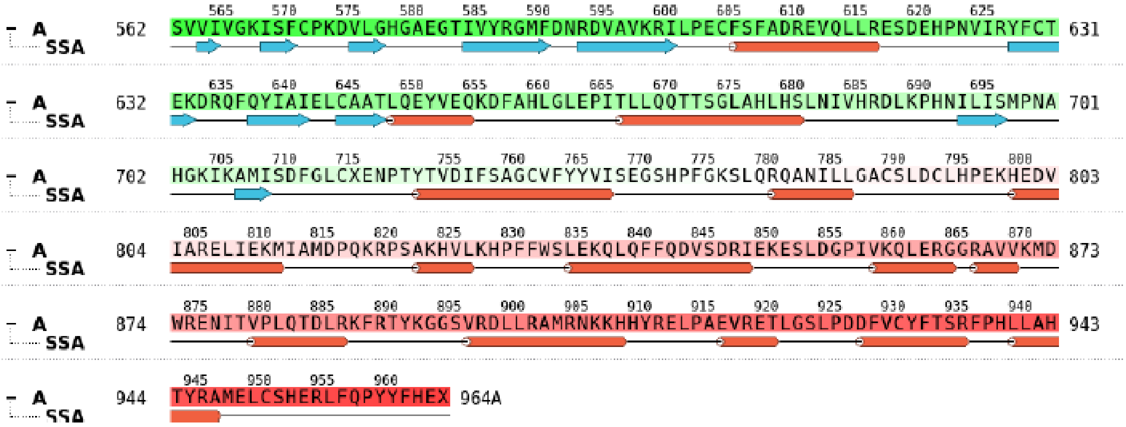


## Ligand Information

| | |
|---|---|
| SMILES | c1cc(C)ccc1CNC(=O)N(C2)CCC[C@]23CCN(C3)Cc4cc(Cl)c(Cl)cc4 |
| PDB Name | '4K7' |
| Num. of Atoms | 59 (total) 30 (heavy) |
| Atomic Mass | 446.424 au |
| Charge | 0 |
| Mol. Formula | C24H29Cl2N3O |
| Num. of Fragments | 5 |
| Num. of Rot. Bonds | 6 |

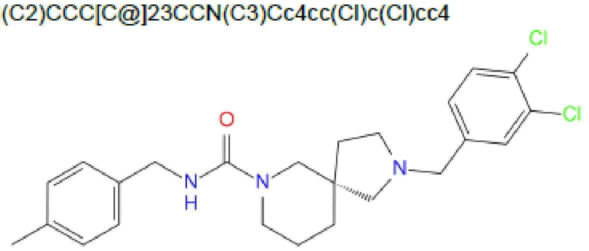


## Counter Ion/Salt Information

| Type | Num. | Concentration [mM] | Total Charge |
|---|---|---|---|
| Na | 134 | 51.082 | +134 |
| Cl | 133 | 50.701 | -133 |

**Figure S14.** MD setup for GSK structure, replica 1.

| CPU # | Job Type | Ensemble | Temp. [K] | Sim. Time [ns] | # Atoms | # Waters | Charge |
|---|---|---|---|---|---|---|---|
| Unknown* | FEP | Unknown* | 300.0 | 500.504 | 149430 | 47695 | 0 |

* The configuration file (-out.cfg) was not found. Keep it in same directory as .aef file.

## Protein Information

| Tot. Residues | Prot. Chain(s) | Res. in Chain(s) | # Atoms | # Heavy Atoms | Charge |
|---|---|---|---|---|---|
| 374 | 'A' | ict_values([374] | 6019 | 3018 | -1 |

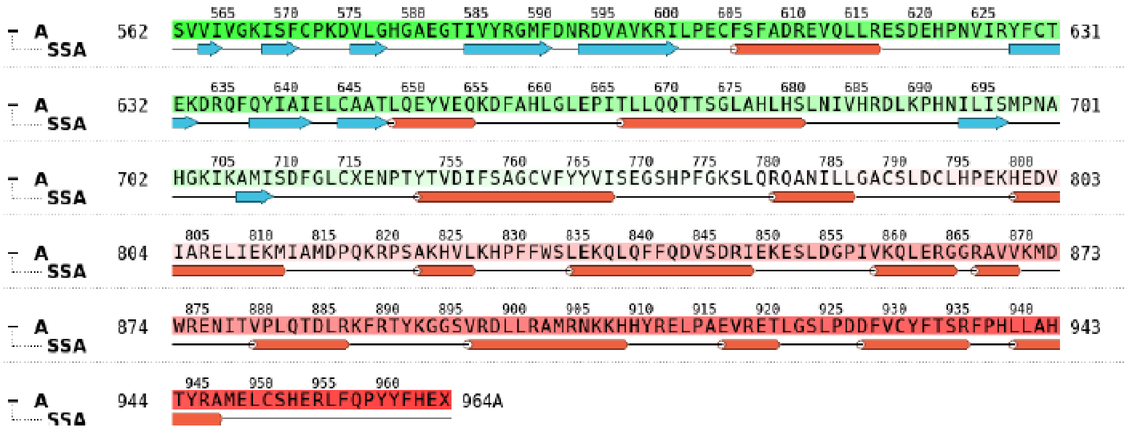


## Ligand Information

| | |
|---|---|
| SMILES | c1cc(C)ccc1CNC(=O)N(C2)CCC[C@]23CCN(C3)Cc4cc(Cl)c(Cl)cc4 |
| PDB Name | '4K7' |
| Num. of Atoms | 59 (total) 30 (heavy) |
| Atomic Mass | 446.424 au |
| Charge | 0 |
| Mol. Formula | C24H29Cl2N3O |
| Num. of Fragments | 5 |
| Num. of Rot. Bonds | 6 |

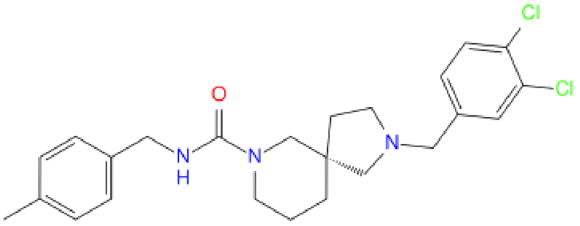


## Counter Ion/Salt Information

| Type | Num. | Concentration [mM] | Total Charge |
|---|---|---|---|
| Na | 134 | 51.082 | +134 |
| Cl | 133 | 50.701 | -133 |

**Figure S15.** MD setup for GSK structure, replica 2.

| CPU # | Job Type | Ensemble | Temp. [K] | Sim. Time [ns] | # Atoms | # Waters | Charge |
|---|---|---|---|---|---|---|---|
| Unknown* | FEP | Unknown* | 300.0 | 500.504 | 149430 | 47695 | 0 |

* The configuration file (-out.cfg) was not found. Keep it in same directory as .aef file.

## Protein Information

| Tot. Residues | Prot. Chain(s) | Res. in Chain(s) | # Atoms | # Heavy Atoms | Charge |
|---|---|---|---|---|---|
| 374 | 'A' | ict_values([374] | 6019 | 3018 | -1 |

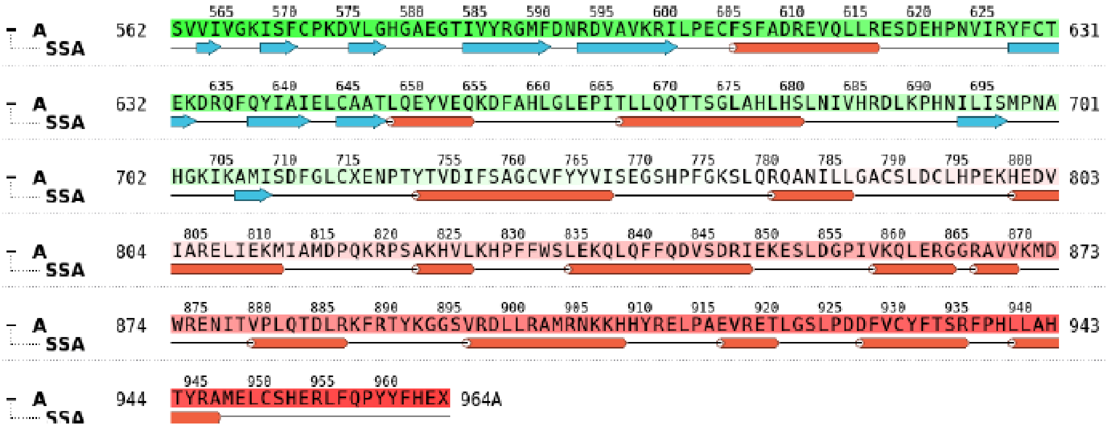


## Ligand Information

| | |
|---|---|
| SMILES | c1cc(C)ccc1CNC(=O)N(C2)CCC[C@]23CCN(C3)Cc4cc(Cl)c(Cl)cc4 |
| PDB Name | '4K7' |
| Num. of Atoms | 59 (total) 30 (heavy) |
| Atomic Mass | 446.424 au |
| Charge | 0 |
| Mol. Formula | C24H29Cl2N3O |
| Num. of Fragments | 5 |
| Num. of Rot. Bonds | 6 |

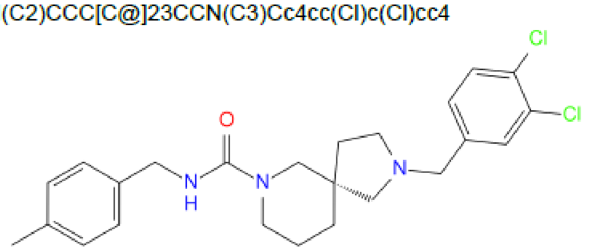


## Counter Ion/Salt Information

| Type | Num. | Concentration [mM] | Total Charge |
|---|---|---|---|
| Na | 134 | 51.082 | +134 |
| Cl | 133 | 50.701 | -133 |

**Figure S16.** MD setup for GSK structure, replica 3.

| CPU # | Job Type | Ensemble | Temp. [K] | Sim. Time [ns] | # Atoms | # Waters | Charge |
|---|---|---|---|---|---|---|---|
| Unknown* | Unknown* | Unknown* | 300.0 | 501.001 | 119763 | 37653 | 0 |

* The configuration file (-out.cfg) was not found. Keep it in same directory as .aef file.

## Protein Information

| Tot. Residues | Prot. Chain(s) | Res. in Chain(s) | # Atoms | # Heavy Atoms | Charge |
|---|---|---|---|---|---|
| 405 | 'A' | ict_values([405] | 6516 | 3265 | +2 |

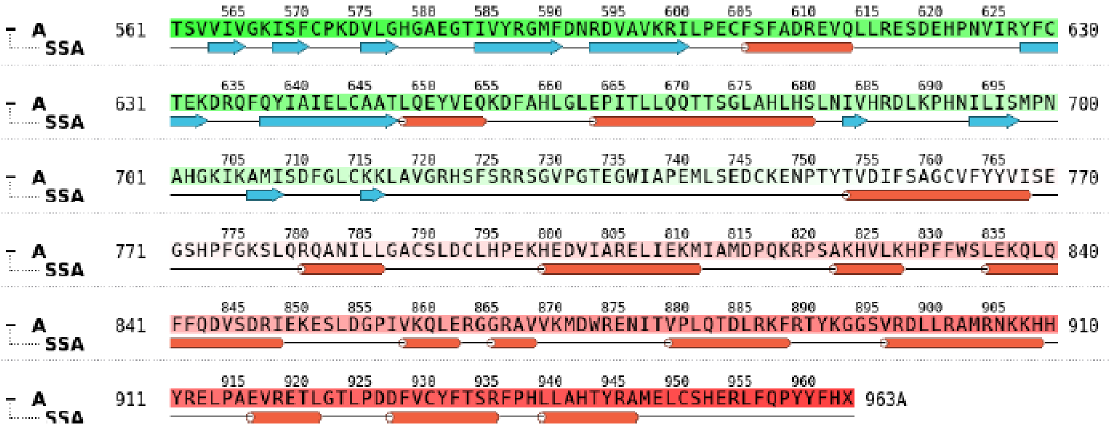


## Ligand Information

| | |
|---|---|
| SMILES | c1cccc(Cl)c1S(=O)(=O)Nc(c(c23)cccc3)cc(C)c2Oc(nccc4)c4-c5ccnc(n5)N[C@H]6CC[C@H]([NH3+])CC6 |
| PDB Name | '3E4' |
| Num. of Atoms | 75 (total) 43 (heavy) |
| Atomic Mass | 616.167 au |
| Charge | +1 |
| Mol. Formula | C32H32ClN6O3S |
| Num. of Fragments | 6 |
| Num. of Rot. Bonds | 8 |

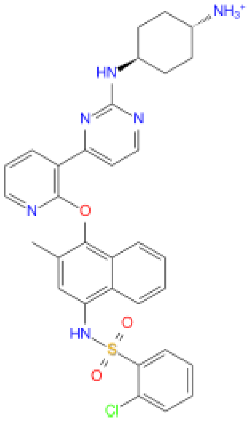


## Counter Ion/Salt Information

| Type | Num. | Concentration [mM] | Total Charge |
|---|---|---|---|
| Cl | 108 | 52.151 | -108 |
| Na | 105 | 50.702 | +105 |

**Figure S17.** MD setup for KIRA structure, replica 1.

| CPU # | Job Type | Ensemble | Temp. [K] | Sim. Time [ns] | # Atoms | # Waters | Charge |
|---|---|---|---|---|---|---|---|
| Unknown* | FEP | Unknown* | 300.0 | 500.504 | 119763 | 37653 | 0 |

* The configuration file (-out.cfg) was not found. Keep it in same directory as .aef file.

## Protein Information

| Tot. Residues | Prot. Chain(s) | Res. in Chain(s) | # Atoms | # Heavy Atoms | Charge |
|---|---|---|---|---|---|
| 405 | 'A' | ict_values([405]) | 6516 | 3265 | +2 |

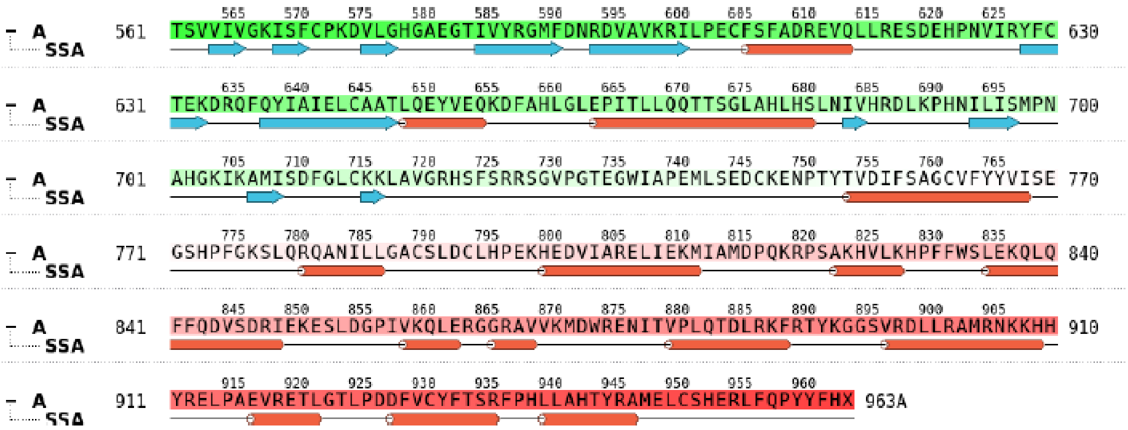


## Ligand Information

| | |
|---|---|
| SMILES | c1cccc(Cl)c1S(=O)(=O)Nc(c(c23)cccc3)cc(C)c2Oc(nccc4)c4-c5ccnc(n5)N[C@H]6CC[C@H]([NH3+])CC6 |
| PDB Name | '3E4' |
| Num. of Atoms | 75 (total) 43 (heavy) |
| Atomic Mass | 616.167 au |
| Charge | +1 |
| Mol. Formula | C32H32ClN6O3S |
| Num. of Fragments | 6 |
| Num. of Rot. Bonds | 8 |

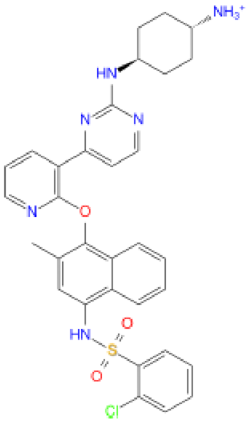


## Counter Ion/Salt Information

| Type | Num. | Concentration [mM] | Total Charge |
|---|---|---|---|
| Cl | 108 | 52.151 | -108 |
| Na | 105 | 50.702 | +105 |

**Figure S18.** MD setup for KIRA structure, replica 2.

| CPU # | Job Type | Ensemble | Temp. [K] | Sim. Time [ns] | # Atoms | # Waters | Charge |
|---|---|---|---|---|---|---|---|
| Unknown* | FEP | Unknown* | 300.0 | 500.504 | 119763 | 37653 | 0 |

* The configuration file (-out.cfg) was not found. Keep it in same directory as .aef file.

## Protein Information

| Tot. Residues | Prot. Chain(s) | Res. in Chain(s) | # Atoms | # Heavy Atoms | Charge |
|---|---|---|---|---|---|
| 405 | 'A' | ict_values([405] | 6516 | 3265 | +2 |

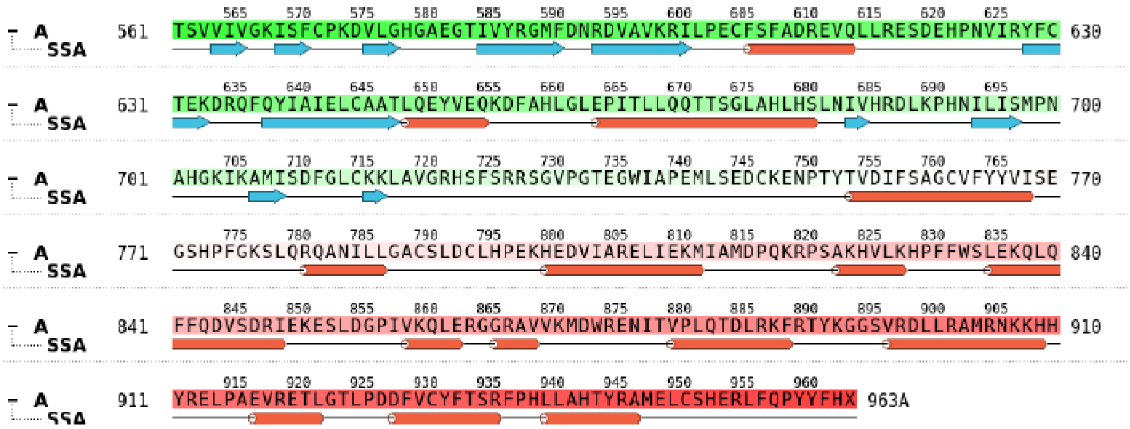


## Ligand Information

| | |
|---|---|
| SMILES | c1cccc(Cl)c1S(=O)(=O)Nc(c(c23)cccc3)cc(C)c2Oc(nccc4)c4-c5ccnc(n5)N[C@H]6CC[C@H]([NH3+])CC6 |
| PDB Name | '3E4' |
| Num. of Atoms | 75 (total) 43 (heavy) |
| Atomic Mass | 616.167 au |
| Charge | +1 |
| Mol. Formula | C32H32ClN6O3S |
| Num. of Fragments | 6 |
| Num. of Rot. Bonds | 8 |

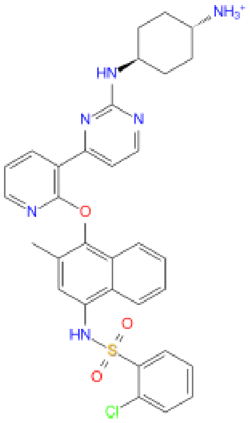


## Counter Ion/Salt Information

| Type | Num. | Concentration [mM] | Total Charge |
|---|---|---|---|
| Cl | 108 | 52.151 | -108 |
| Na | 105 | 50.702 | +105 |

**Figure S19.** MD setup for KIRA structure, replica 3.

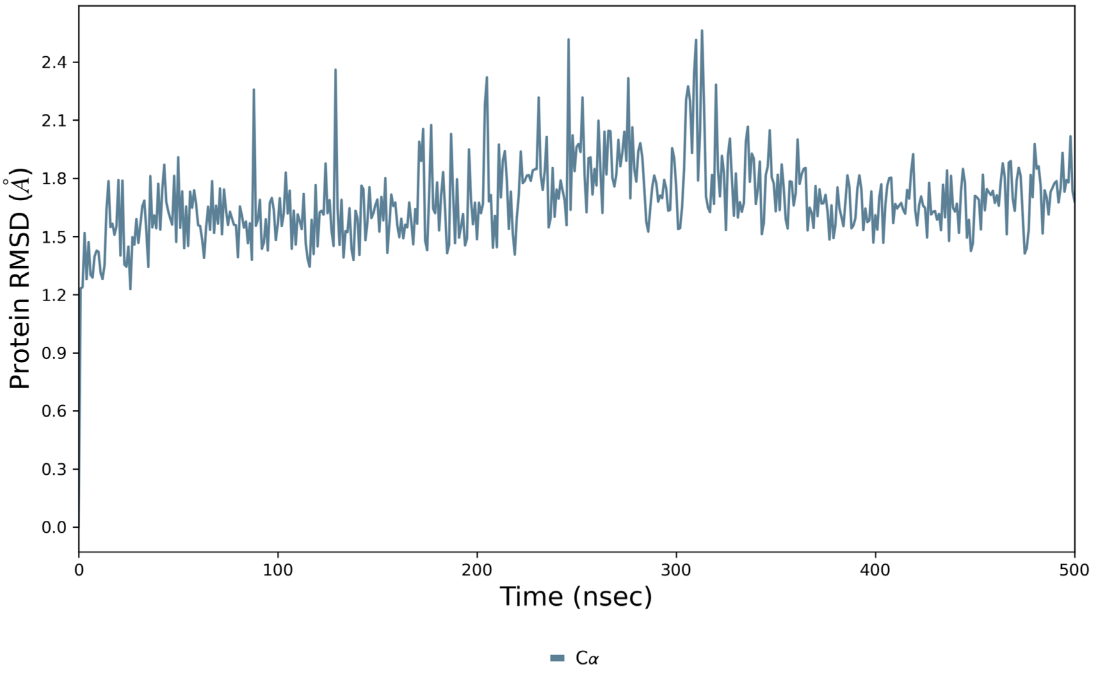


**Figure S20.** Cα rmsd plot for MD simulation of apo structure, replica 1.

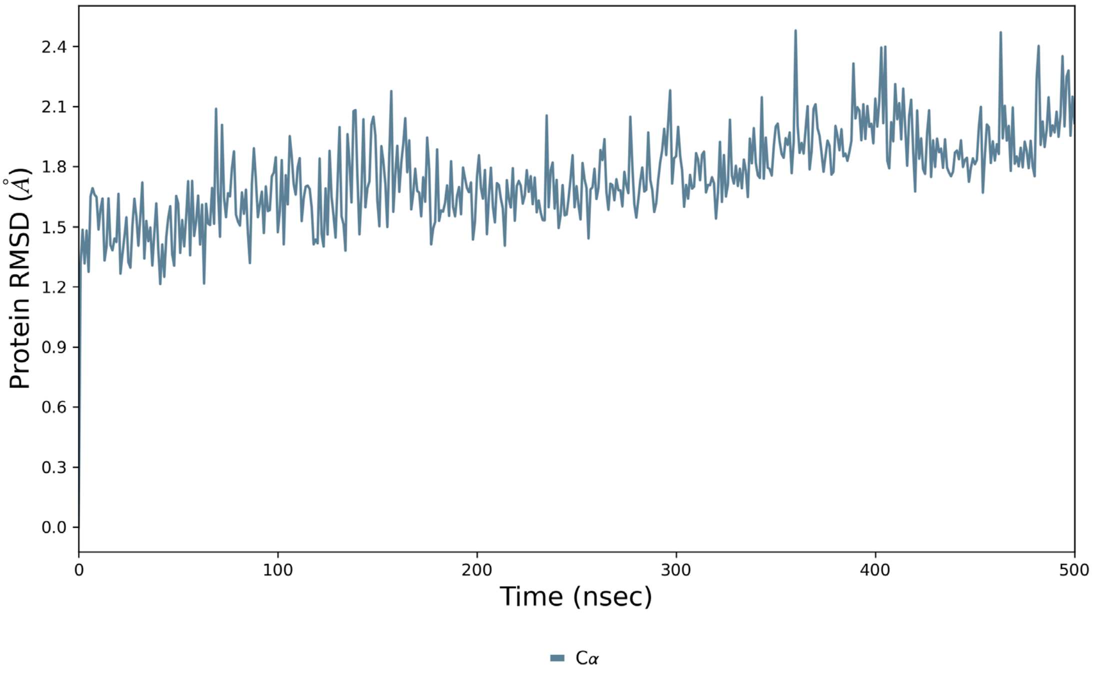


**Figure S21.** Cα rmsd plot for MD simulation of apo structure, replica 2.

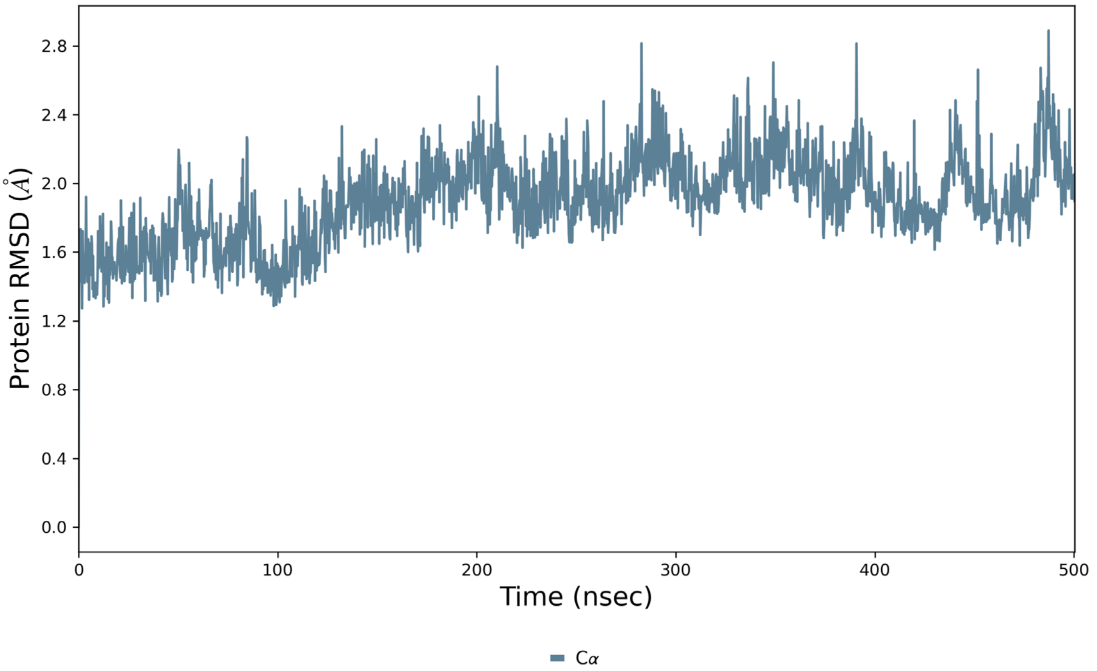


**Figure S22.** Cα rmsd plot for MD simulation of apo structure, replica 3.

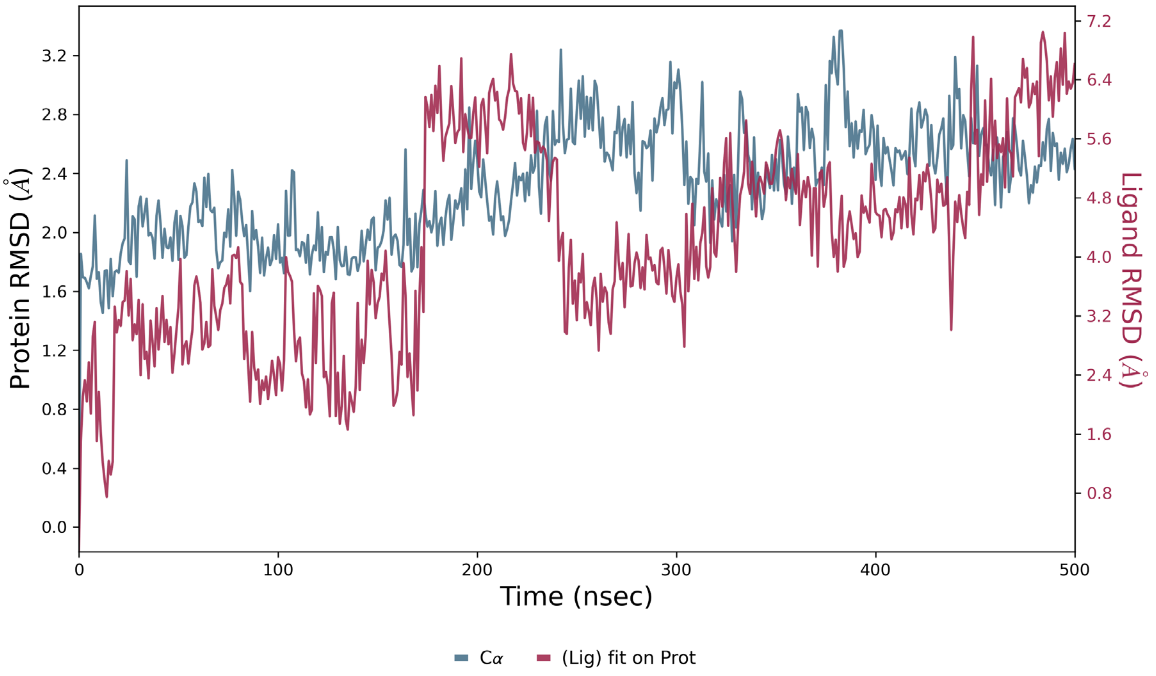


**Figure S23.** Cα and ligand rmsd plot for MD simulation of GSK structure, replica 1.

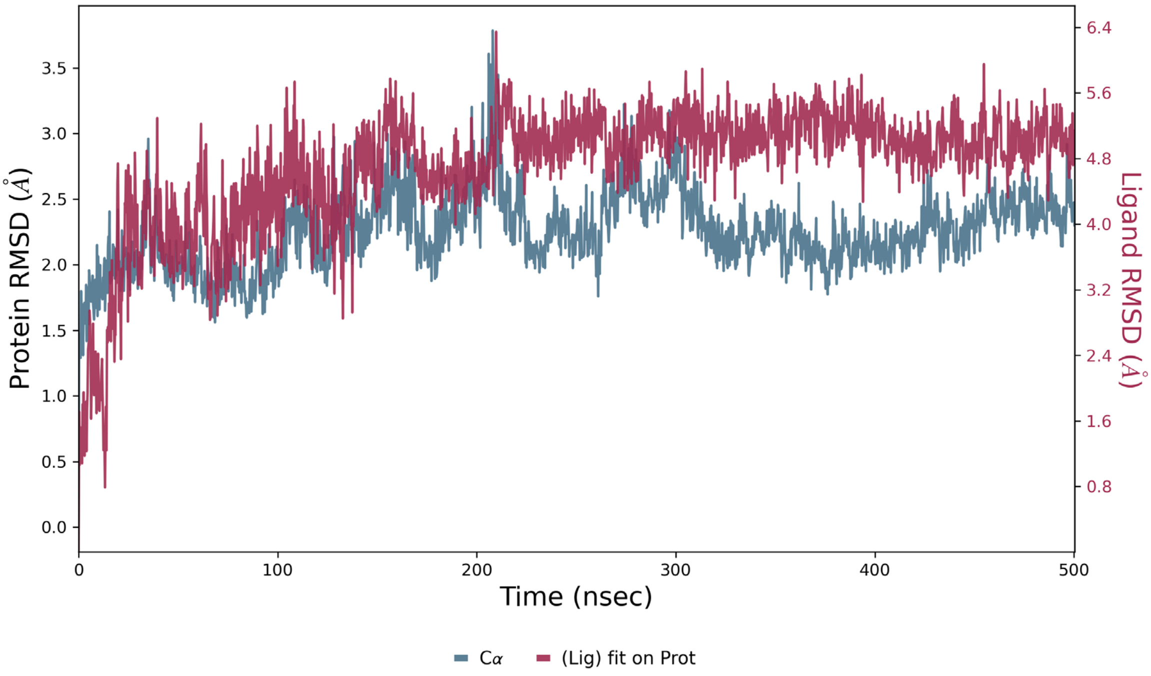


**Figure S24.** Cα and ligand rmsd plot for MD simulation of GSK structure, replica 2.

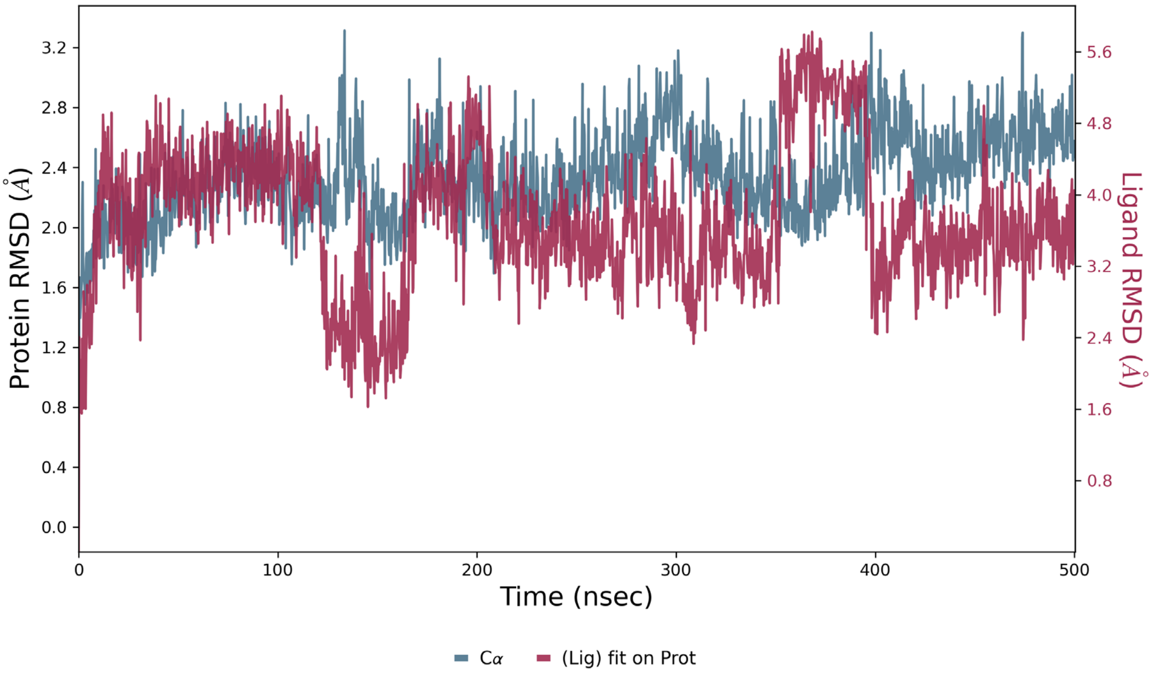


**Figure S25.** Cα and ligand rmsd plot for MD simulation of GSK structure, replica 3.

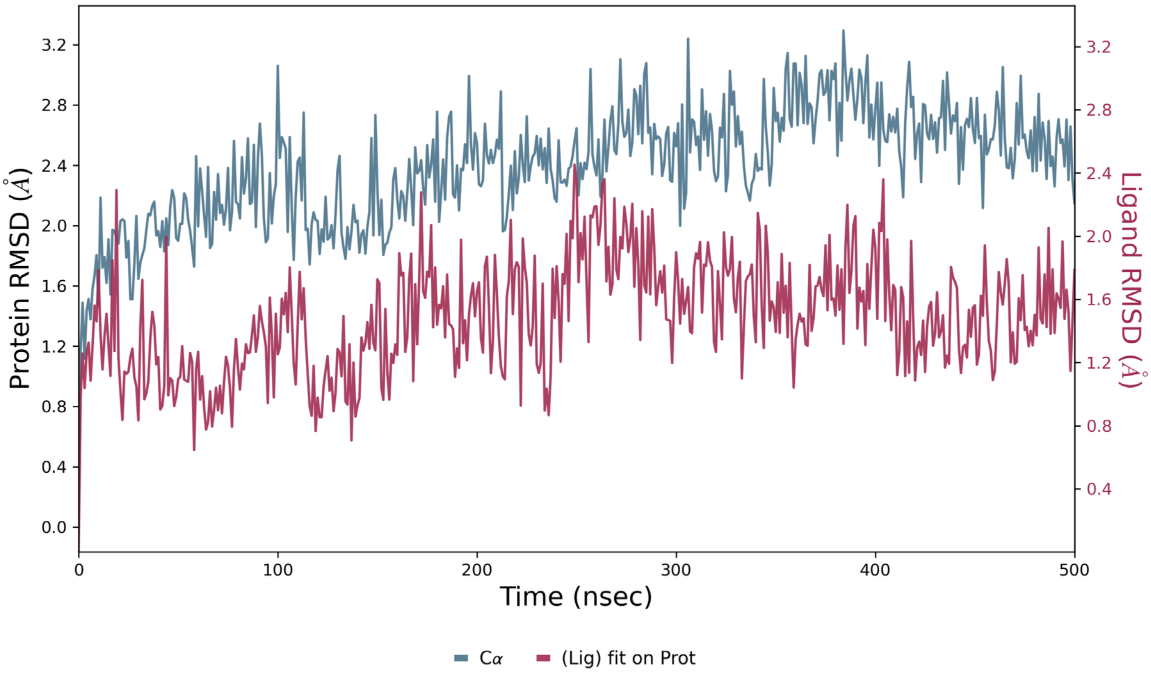


**Figure S26.** Cα and ligand rmsd plot for MD simulation of KIRA structure, replica 1.

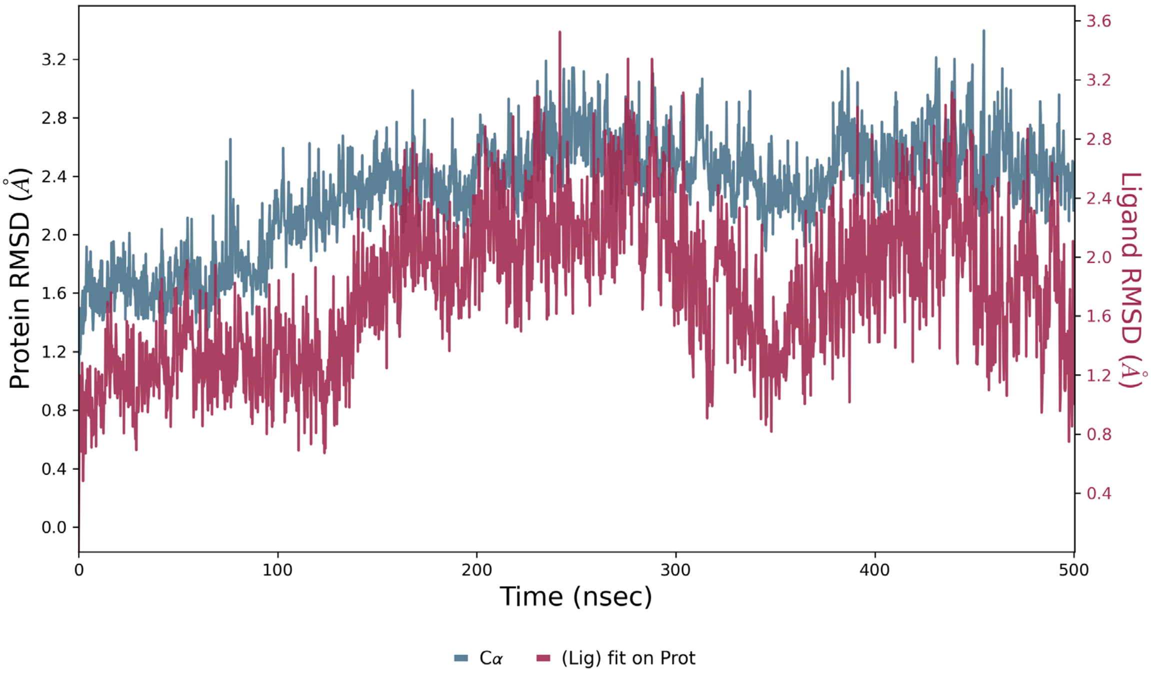


**Figure S27.** Cα and ligand rmsd plot for MD simulation of KIRA structure, replica 2.

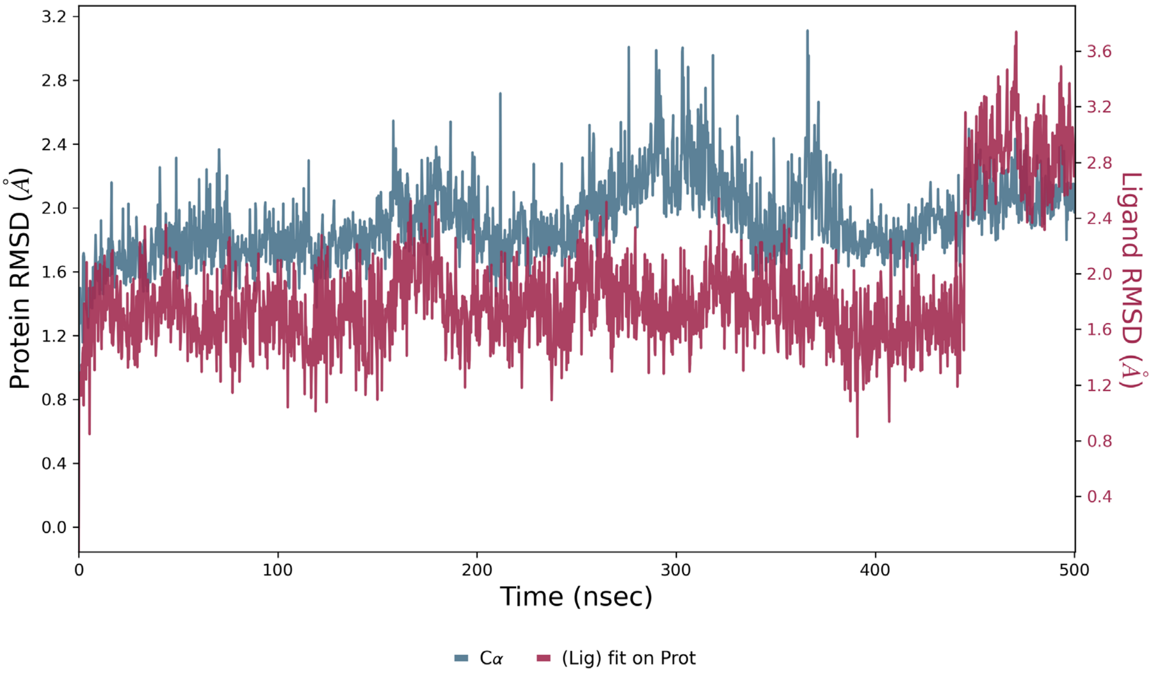


**Figure S28.** Cα and ligand rmsd plot for MD simulation of KIRA structure, replica 3.

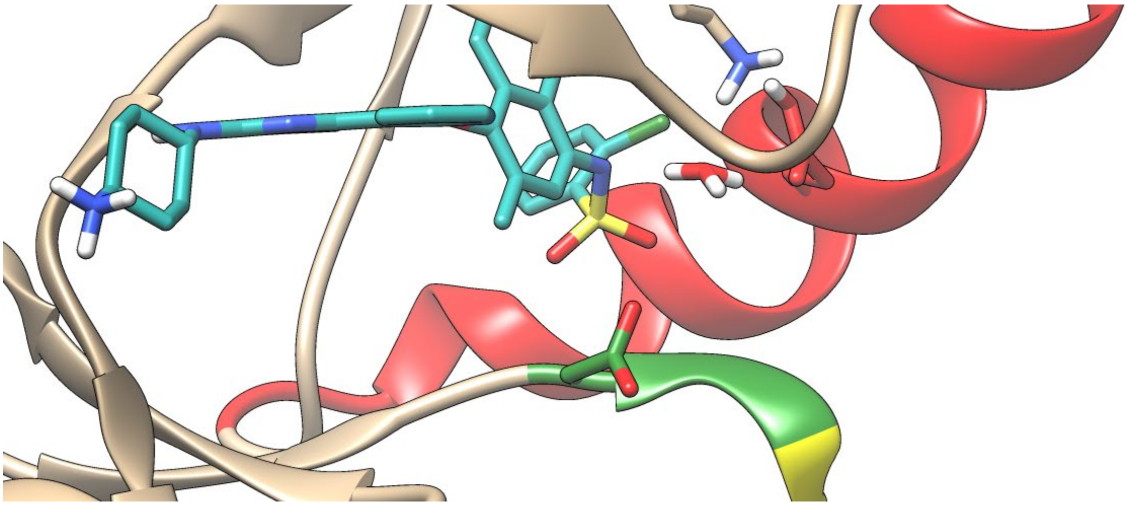

**Figure S29.** The water in the active site attracts the proton of the sulfonamide nitrogen in KIRA, thus forming $SO_2N^-$ + $H_3O^+$ during the energy optimization.

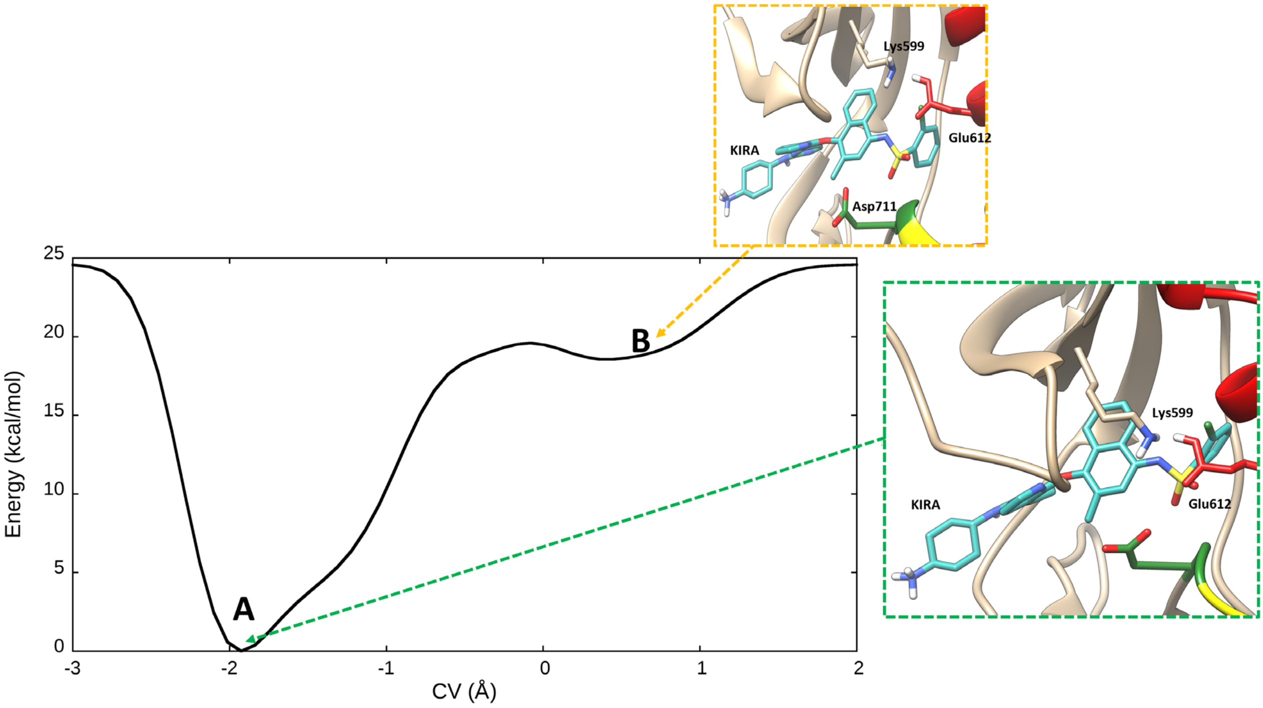


**Figure S30.** 1D projected FES CV1 of the KIRA system (PDB code:4U6R). The local structures at the energetic minima are shown as inserts.

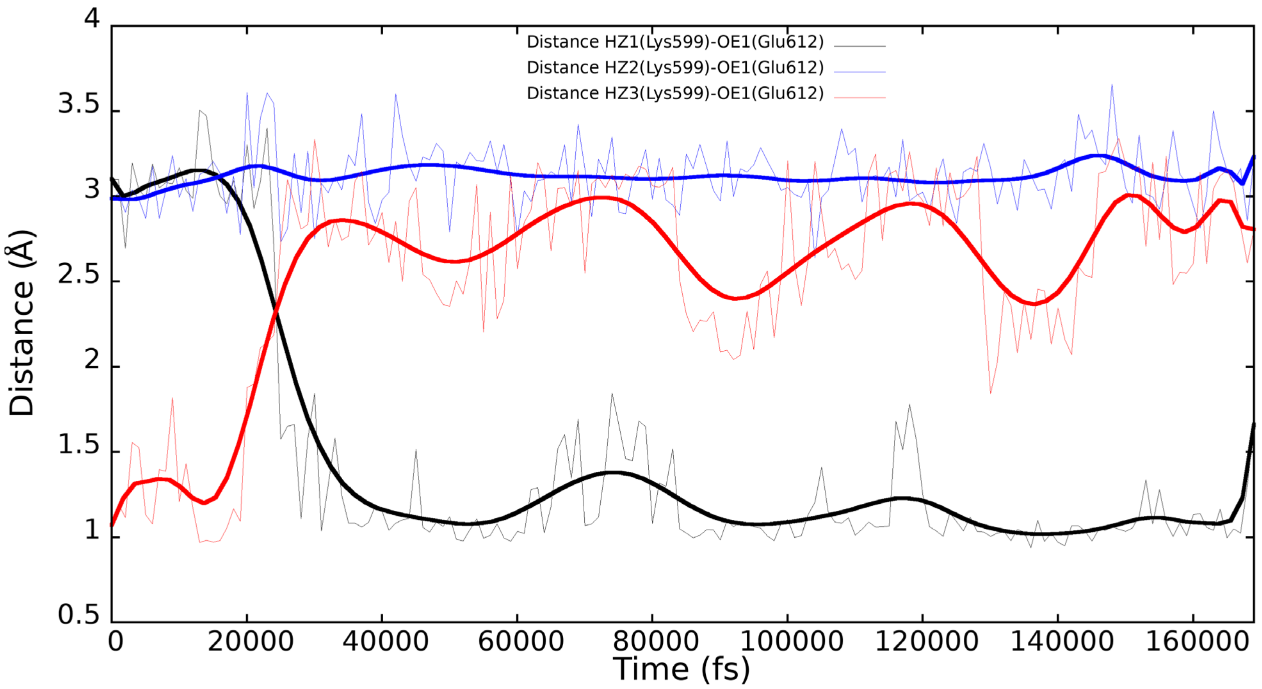


**Figure S31.** The evolution of the distance between OE1 (carboxylate oxygen) of Glu612 and the terminal protons in the Lys599 protonated amine group during the QMMM WT-MetaD.

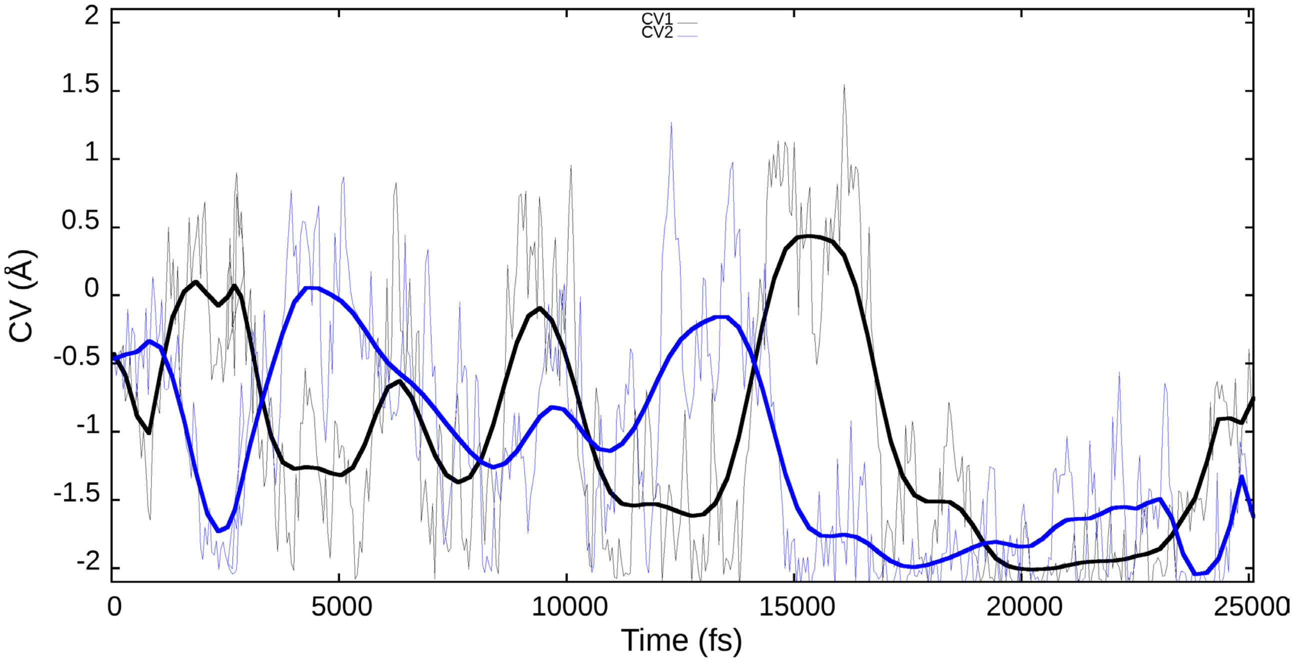


**Figure S32.** Recrossing event *i.e.* charged state-neutral state-charged state occurred after 25 ps for apo state of IRE1 (PDB code: 6W3B).

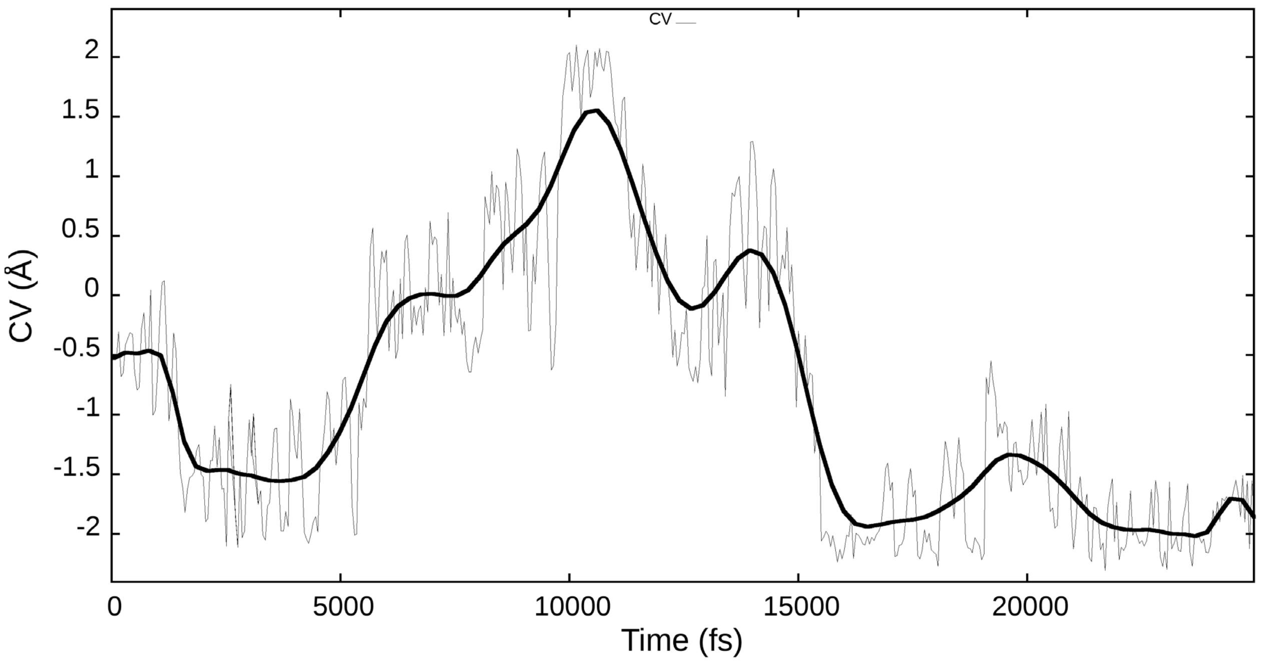


**Figure S33.** Recrossing event *i.e.* charged state-neutral state-charged state occurred after 25 ps for GSK (PDB code: 4YZ9).

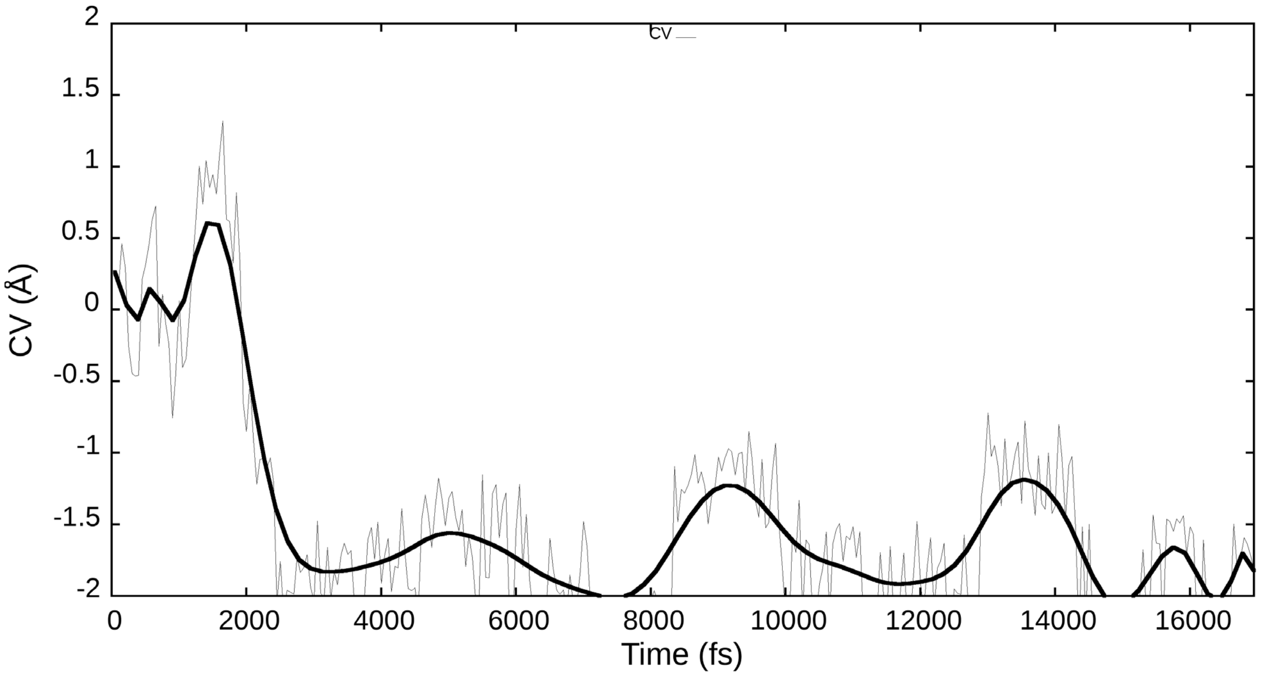


**Figure S34.** Recrossing event *i.e.* charged state-neutral state-charged state occurred after 17 ps for KIRA (PDB code: 4U6R).

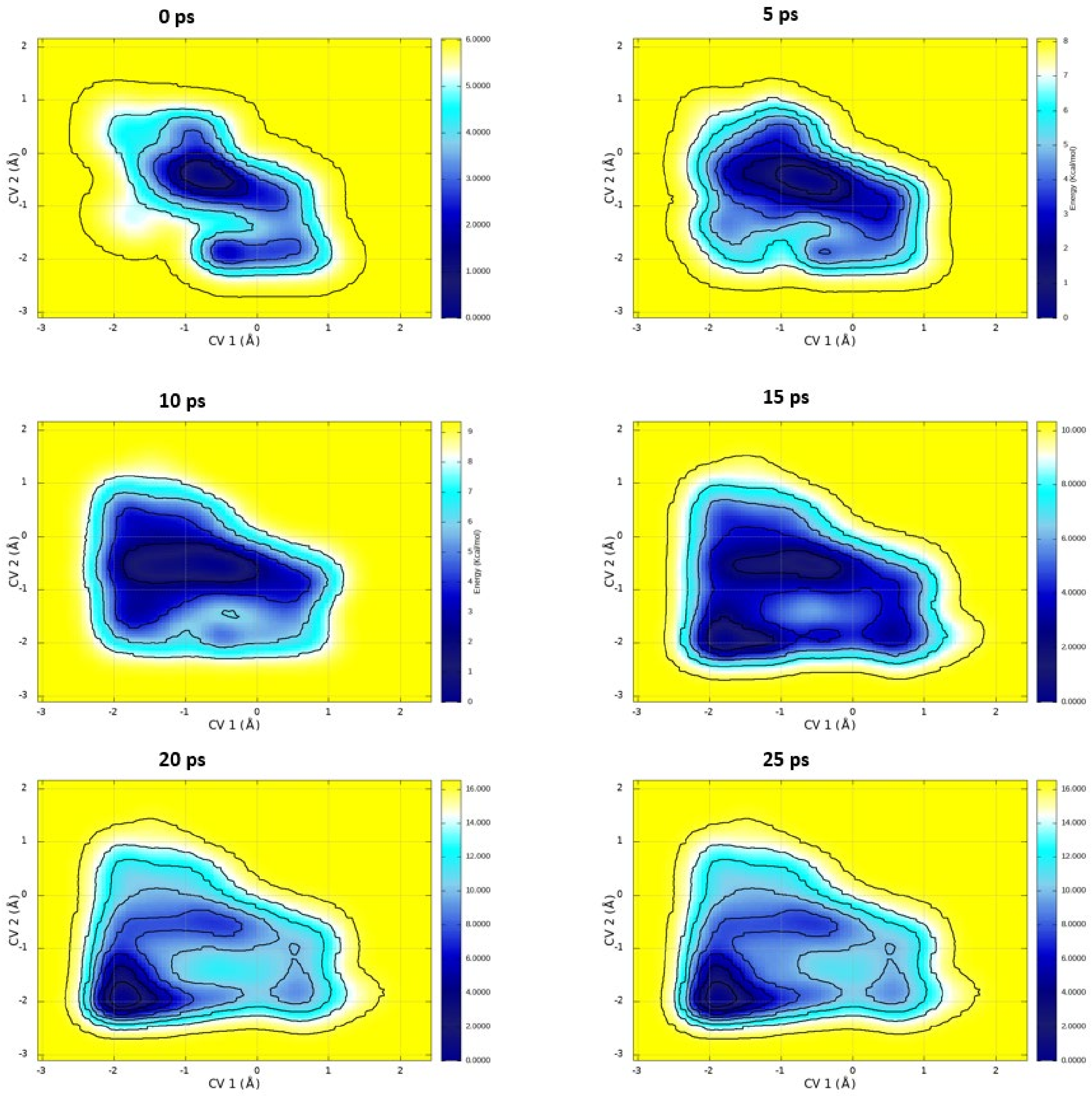


**Figure S35.** Free Energy Surface (FES) check as function of simulation time for the apo state of IRE1 (PDB code: 6W3B).

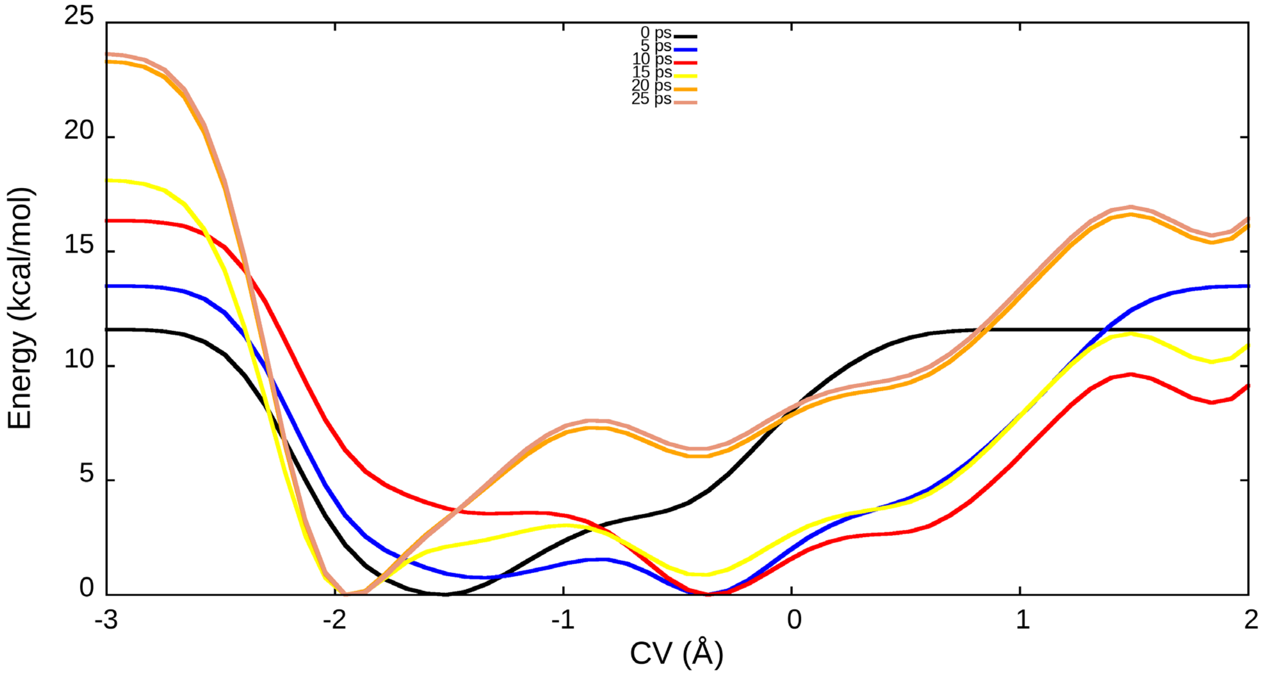


**Figure S36.** Variation in CV1 along the Free Energy Surface (FES) at different simulation times for GSK (PDB code: 4YZ9).

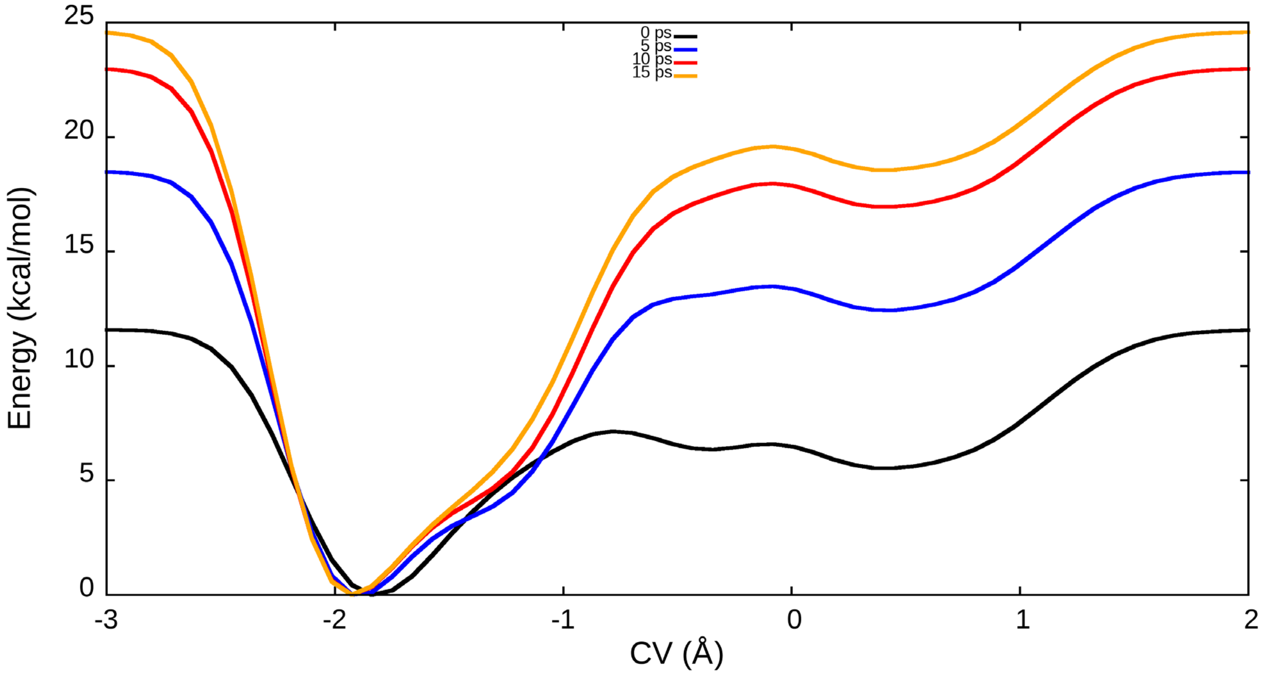


**Figure S37.** Variation of CV1 along the Free Energy Surface (FES) at different simulation times for KIRA (PDB code: 4U6R).

.
